# Human iPS derived progenitors bioengineered into liver organoids using an inverted colloidal crystal poly (ethylene glycol) scaffold

**DOI:** 10.1016/j.biomaterials.2018.07.043

**Published:** 2018-11

**Authors:** Soon Seng Ng, Kourosh Saeb-Parsy, Samuel J.I. Blackford, Joe M. Segal, Maria Paola Serra, Marta Horcas-Lopez, Da Yoon No, Sotiris Mastoridis, Wayel Jassem, Curtis W. Frank, Nam Joon Cho, Hiromitsu Nakauchi, Jeffrey S. Glenn, S. Tamir Rashid

**Affiliations:** a Centre for Stem Cells and Regenerative Medicine & Institute for Liver Studies, King's College London, England, UK; b Division of Gastroenterology and Hepatology, Department of Medicine, Stanford University School of Medicine, Stanford, CA, USA; c Department of Microbiology and Immunology, Stanford University School of Medicine, Stanford, CA, USA; d Department of Surgery, University of Cambridge and the Cambridge NIHR Biomedical Research Centre, Cambridge, UK; e Department of Bioengineering, Stanford University, Stanford, CA, USA; f Department of Chemical Engineering, Stanford University, Stanford, CA, USA; g School of Materials Science and Engineering, Nanyang Technological University, Singapore, Singapore; h Institute for Stem Cell Biology and Regenerative Medicine, Stanford University School of Medicine, Stanford, CA, USA

**Keywords:** Biomimetic materials, Liver stem cells, Bioengineering, Organogenesis, iPSC, induced pluripotent stem cell, IH, iPSC derived hepatic progenitors, FDA, food and drug administration, TGFβ, transforming growth factor beta, 3D, three-dimensional, ICC, inverted colloidal crystal, PEG, polyethylene glycol, ECM, extracellular matrix, Col, collagen, Fn, fibronectin, Ln, laminin, CK19, keratin 19, ECAM, epithelial cell adhesion molecule, AFP, alpha-fetoprotein, ASGR1, asialoglycoprotein receptor 1, ALB, albumin, HNF4a, hepatocyte nuclear factor 4-alpha, RT-qPCR, reverse transcription-quantitative polymerase chain reaction, 2D, two-dimensional, RNA-seq, RNA-sequence, DE, iPSC derived definitive endoderm, PCA, principle component analysis, MRP2, multidrug resistance protein 1, BSEP, bile-salt efflux pump, ZO-1, tight junction protein 1, CLF, cholyl-lysyl-flourescence, TGZ, Troglitazone, HCV, hepatitis C virus, CLDN1, claudin 1, OCLN, occludin, SCARB1, scavenger receptor class B type 1, LDLR, low-density lipoprotein receptor, HSGP, heparin sulfate glycoprotein, H&E, haematoxylin and eosin, CYC, cyclopamine, HUVEC, human umbilical endothelial cell, MSC, mesenchymal stem cell

## Abstract

Generation of human organoids from induced pluripotent stem cells (iPSCs) offers exciting possibilities for developmental biology, disease modelling and cell therapy. Significant advances towards those goals have been hampered by dependence on animal derived matrices (e.g. Matrigel), immortalized cell lines and resultant structures that are difficult to control or scale. To address these challenges, we aimed to develop a fully defined liver organoid platform using inverted colloid crystal (ICC) whose 3-dimensional mechanical properties could be engineered to recapitulate the extracellular niche sensed by hepatic progenitors during human development. iPSC derived hepatic progenitors (IH) formed organoids most optimally in ICC scaffolds constructed with 140 μm diameter pores coated with type I collagen in a two-step process mimicking liver bud formation. The resultant organoids were closer to adult tissue, compared to 2D and 3D controls, with respect to morphology, gene expression, protein secretion, drug metabolism and viral infection and could integrate, vascularise and function following implantation into livers of immune-deficient mice. Preliminary interrogation of the underpinning mechanisms highlighted the importance of TGFβ and hedgehog signalling pathways. The combination of functional relevance with tuneable mechanical properties leads us to propose this bioengineered platform to be ideally suited for a range of future mechanistic and clinical organoid related applications.

## 1 Introduction

Induced pluripotent stem cells offer exciting new possibilities in developmental biology, disease modelling and transplantation. Comprehensive realization of that promise is likely to require consolidation with other emerging technologies such as 3D cell culture. Along those lines, hepatic progenitor cells derived from iPSCs [1] and primary tissue [2] were recently shown to form “Organoids” (self-organising miniaturized 3D structures resembling liver tissue) following 3D culture initiated by Matrigel. Organoid generation using Matrigel has similarly been demonstrated across a broad range of tissue types including intestine, kidney and brain [[3], [4], [5]], underlining the importance of this novel approach across the stem cell field. Downstream applications are however limited by the use of Matrigel which is poorly characterized, highly variable and of mouse origin [6]. A bioengineered substitute is therefore essential and was recently reported for intestinal organoid generation [7]. A similar bottom up engineering approach for liver organoid production is urgently needed. We recently developed a novel hepatocyte culture system composed of a 3D-hexagonally arrayed inverted colloidal crystal (ICC) scaffold [8]. In comparison to other 3D culture systems, the ICC boasts several advantages. These include being made from an FDA approved material, polyethylene glycol (PEG), that can be functionalized with select ECM proteins or varied in mechanical stiffness, a highly uniform architecture which incorporates size-selectable pores to facilitate tissue interconnection across the whole module whilst retaining uniform nutrient penetration to all cells and finally, being transparent to provide an easy means of viewing cells and intra-cellular fluorescence over time. Exploiting these properties, we were able to facilitate induction of organoid structures with advanced liver function in primary human fetal liver cells cultured in ICC's [9]. Lack of donor material, ethical constraints and biological heterogeneity however make further progress difficult with human fetal cells. We therefore turned to the recently established technology of human iPSC-derived hepatocytes [10,11], as a good biological approximation to human fetal liver cells but not limited by the same constraints cited above, to explore their potential for organoid generation.

## 2 Materials & methods

### 2.1 Study design

The overall objective of this study was to test whether organoids bioengineered in ICC scaffolds exhibited a more physiologically relevant liver phenotype compared to conventional 2D or 3D (Matrigel and Spheroid) models. iPSC-derived hepatic progenitors (IH) differentiated from the same parental line were therefore matured in four different in vitro models (ICC, 3D Matrigel, Spheroid and 2D) for 14 days and compared. Measurement techniques to compare the three models were designed to characterize two major endpoints: morphology (organoid formation) and function (gene expression, protein expression, liver specific functions and in vivo behavior). In transplant experiments, littermate animals were randomly assigned to experimental groups and analysis of samples was performed blindly. Studies were repeated at least three times.

### 2.2 Cells

All human tissues were collected with informed consent following ethical and institutional guidelines. Freshly isolated hepatocytes were obtained from Triangle Research Labs (TRL) and Human Developmental Biology Resource (HDBR) University College London (UCL). Fetal hepatocytes were obtained from 16 to 20 week old fetuses (HDBR), dissociated as previously described [12]. Three different human iPSC lines were used to generate hepatic progenitors (IH) for these experiments: [1,10] (https://stemcells.nindsgenetics.org/), [11] the latter of which is considered to be of ‘clinical grade’ and thereby potentially suitable for future human therapy.

### 2.3 ICC fabrication

**Fig. 1:**
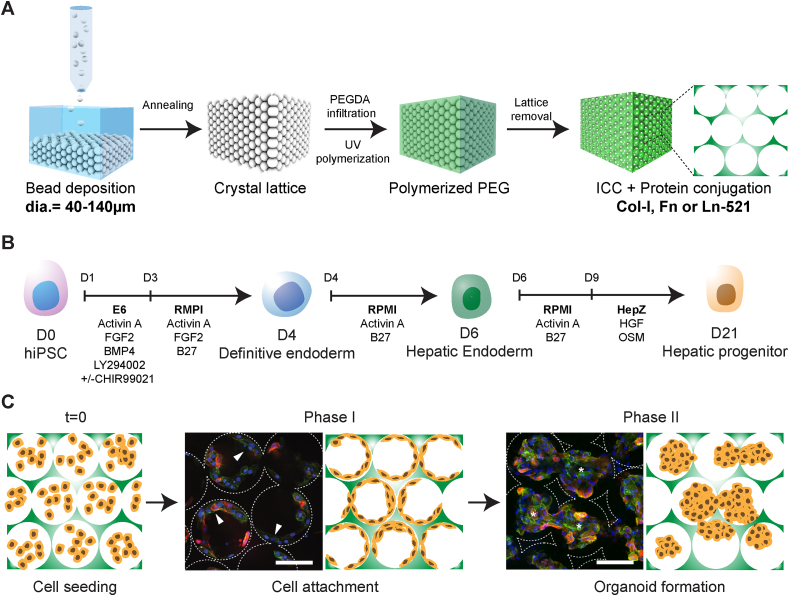
Bioengineering liver organoids using ICC scaffold and iPSC-derived hepatic progenitor (IH). (**A**) Schematic illustration of ICC fabrication using a range of sacrificial monodispersed beads and ECM proteins for stem cell niche modulation. (**B**) Schematic of human iPSC-derived hepatic progenitors differentiation protocol. (**C**) Schematic of bioengineering liver organoids using IH and ICC in three main steps, cell seeding, cell attachment (Phase I) and organoid formation (Phase II). Confocal micrographs of human fetal liver cells (Fetal; CTNNB green; CK19 red) demonstrating the two-phase organoid formation in Col-I coated ICC with 140 μm pore size. Arrowheads indicate cells lining surface of ICC; asterisks represent cells forming clusters. Scale bar, 100 μm. (For interpretation of the references to colour in this figure legend, the reader is referred to the Web version of this article.)

Sacrificial crystal lattices were constructed on a rhombus PDMS mold with 17mmx15 mm diagonal lengths and 2 mm depth using polystyrene beads with diameter of 40 μm, 60 μm, 100 μm, or 140 μm (Duke Scientific) and annealed under 140 °C for 1.5 h. Lattices were then infiltrated with precursor solution of 50 %w/v polyethylene glycol-diacrylate (PEGDA, Alfa Aesar), 10 %w/v acrylate-PEG-N-hydroxysuccinimide (Laysan Bio Inc, AL) and 1 %w/v photoinitiator (Irgacure^®^ 2959, BASF, Switzerland) in DI water. The precursor solution underwent free-radical induced polymerization under 75 W xenon ultraviolet (UV) light source (Oriel Instruments, Mountain View, CA) for 10 min. After polymerization, the polystyrene sacrificial lattice was removed by tetrahydrofuran treatment for 2 h. The resulting ICC hydrogel scaffolds were equilibrated in deionized water and conjugated with appropriate ECM proteins, such as 100 μg/mL Collagen I (Col-I, Sigma), 50 μg/mL Fibronectin (Fn, RnD), and 10 μg/mL Laminin 521 (Ln-521, Biolamina) (Fig. 1A). The final tubular ICC scaffolds for 96 well plate format culture were created using 6 mm biopsy punch.

### 2.4 iPSC-derived liver organoid culture

iPSC-derived hepatic progenitors (IH) were generated using our established protocol (Fig. 1B) [13]. To generate iPSC liver organoids, we suspended approximately 0.5 × 10^6^ IH cells in Hepatozyme medium (Life Technologies), supplemented with Oncostatin M 0.01 μg/ml (Peprotech) and Hepatocyte Growth Factor 0.05 μg/ml (Peprotech) with the final cell density of 125 × 10^6^ cells/ml (Fig. 1C). Approximately 4 μl cell suspension was then pipetted onto top and bottom surfaces of partially dehydrated ICC scaffolds and placed in an incubator for 30 min without media to minimize cell loss and maximize cell attachment. Cell-laden ICC scaffolds were then transferred to a new 96-well plate for culture and media were refreshed at 4 h post-seeding to remove excessive cells that are not attached to surface or established cellular clusters. Cells were left for a further 14 days to mature. Media was refreshed every two days. 2D controls were generated using IH cells seeded into standard 48 well plate tissue culture plastic plates coated with the same ECM proteins and cultured with the same reagents as used for ICC culture.

### 2.5 Generation of IH-spheroids

A single cell suspension of IH was prepared and 0.3 × 10^6^ cells were seeded per well of a 24-well Aggrewell-400 (STEMCELL Technologies, Vancouver, Canada). Aggrewell plates were prepared as recommended by the supplier. Centrifugation at 200G for 3 min was carried out to deposit cells into the microwells of the plate.

### 2.6 Immunofluorescence staining and imaging

After fixation with 4% paraformaldehyde, cells were blocked and permeabilized in 1% w/v bovine serum albumin (BSA, Sigma-Aldrich), 10% donkey serum (Life Technologies) and 0.1% Triton) for 30 min at RT. For nuclear antigens, cells were treated with 0.5% Triton (Sigma-Aldrich), then primary antibodies, CTNNB (mouse, 1:100; bdbiosciences), CK19 (rabbit, 1:500; abcam), HNF4a (rabbit, 1:100; abcam), AFP (mouse, 1:100; abcam) and ALB (goat, 1:100; Bethyl), ASGR1 (mouse, 1:100; Thermo Fisher Scientific), COL1 (rabbit, 1:500; abcam), Vimentin (rabbit, 1:200; abcam); CD44 (mouse, 1:200 abcam), CD31 (rabbit, 1:50; abcam); Col-X (mouse, 1:250; abcam), MRP2 (mouse, 1:200; abcam), ZO-1 (mouse, 1:100; abcam), CD26 (rabbit, 1:100; abcam), Pan-Cadherin (mouse, 1:100; Sigma), Claudin 1 (rabbit, 1:100; abcam), Occludin (mouse, 1:100; abcam), Stem121 (mouse, 1:100; Cellartis), phosphor-SMAD2/3 (rabbit, 1:100; Cell signalling technology) and GLI1 (rabbit, 1:100; abcam) were applied for overnight at 4 °C. After washes, cells were then incubated with Alexa 647, Alexa 568, Alexa 488 conjugated secondary antibodies or 488-Phalloidin (1:250; Life Technologies). Samples were counterstained with DAPI (NucBlue, Life Technologies). Confocal micrographs were captured using Nikon Ti spinning disk confocal microscope equipped with Andor Neo camera and images were processed by NIS-element software.

### 2.7 Bright field imaging

Bright field imaging was captured using Leica DMIL LED equipped with Leica DFC3000 G camera and images processed by LAS X software.

### 2.8 Live/dead staining

Fluorescine diacete (FDA) (Sigma-Aldrich, St. Louis, USA) and cell-impermeant ethidium homodimer-1 (EthD-1) (Thermo Fisher Scientific, Waltham, USA) were used as recommended by the supplier for staining of viable and dead cells. Spheroids and alginate encapsulated cells were incubated in 4 μM EthD-1 for 35 min, washed with Hank's Balanced Salt Solution (HBSS) containing calcium (Thermo Fisher Scientific, Waltham, USA), then incubated in 50 μg/ml FDA for 90 s, and finally washed 5 times with HBSS before imaging.

### 2.9 Cell number quantification

Double stranded DNA was collected, quantified and interpolated from standard curve plotted using a range of known cell number as illustrated by Quant-iT™ PicoGreen™ dsDNA Assay Kit (ThermoFisher Scientific).

### 2.10 Human albumin enzyme-linked immunosorbent assay (ELISA)

Albumin secretion of all different cell types was assessed using the Human Albumin Quantitation Set (Bethyl Laboratories Inc) following manufacturer's instructions.

### 2.11 Immuno-histochemistry staining and imaging

IH-ICC or animal explants were fixed in 10% formalin buffer saline for two days then dehydrated and paraffin wax infiltrated using Excelsior™ AS Tissue Processor. After embedded, sectioned (5 μm), and stained using Mouse and Rabbit Specific HRP/AEC (ABC) Detection IHC Kit (abcam) using antibody CK19 (rabbit, 1:200; abcam), EPCAM (mouse, 1:200; abcam), ALB (mouse, 1:100; abcam) and CD31 (mouse, 1:100; Dako) then counterstained with haematoxylin (abcam). Mounted slides were imaged using NanoZoomer (Hamamatsu).

### 2.12 Image analysis

To analyse the confluency and cluster morphology of cells in ICC, selected images of F-actin staining were reconstructed using maximum intensity projection. The confluency index =(*Cc/Cp × 100%*) is introduced to quantify the proportion of the ICC surface covered by cell attachment by measuring the circumference of cell lining (*Cc*) over the overall circumference of pores in ICC (*Cp*). The cluster index = (*Kc/Kp × 100%*) is introduced to quantify the number of cell cluster in each pore (*Kc*) against the total number of pores (*Kp*) in the frame.

### 2.13 Fluorescence-activated cell sorting (FACS) staining and analysis

Cells were dissociated from organoid or 2D culture using TrypLE reagent (Thermo Fisher Scientific) and stained using the same condition as immunofluorescence staining protocol stated above. Flow analysis performed using FACSCanto II (BD) and analyized using FlowJo (LLC).

### 2.14 Quantitative real-time PCR (RT-qPCR) analysis

Total RNA was harvested using TRIZOL reagent (Sigma), treated with DNase (Promega) and phenol/chloroform purified. For each sample 0.5 μg of total RNA was reverse transcribed using SuperScript VILO cDNA Synthesis kit (Thermo Fisher Scientific). A typical RT-PCR reaction contained 10 ng of sample cDNA, 0.0075 μl of individual forward and reverse primer each at 100 μM stock, 5 μl Taqman Universal Master mix (Applied Biosystems), 1 μl Taqman target probe (supplementary Figs. 5) and made up to 10 μl with nuclease-free water. Real time PCR reactions were amplified for 40 cycles on a CFX384 Touch™ Real-Time PCR Detection System (Biorad) in triplicate and normalized to ACTB in the same run.

### 2.15 Heat map generation, gene set enrichment analysis, functional network analysis and top canonical pathway analysis

Heat maps were generated from Bulk RNAseq data collected from three Human Fetal Livers, three Human Adult Livers and three iPSC-derived hepatocytes harvested at definitive endodermal stage from the BOB cell line. The three fetal livers included 14pcw, 16pcw and 20pcw. The three adult Livers were Female 18yrs, Male 43yrs and Male 13yrs. RNA was extracted using TRI reagent. Starting with 1 μg input total RNA, Ribosomal RNA was removed using Ribo-Zero Gold rRNA Removal kit (Illumia). Sequencing libraries were prepared using NEBNext^®^ Ultra™ Directional RNA Library Prep Kit for Illumina (N.E.B) using 100 ng rRNA depleted sample and sequenced on a HiSeq 2500 system in Rapid run mode (Illumina) following a standard protocol. All libraries generated between 15 and 25 M reads. Reads were mapped to GRCh38 reference genome using Bowtie2. Raw counts and normalized gene expression was generated using HT-Seq and DESEq2 packages respectively. Heat map was generated using R (http://www.R-project.org) (R Development Core Team, 2008). Heat maps represent average DESEq2 normalized gene expression values of three independent biological samples. Gene set enrichment analysis was performed on normalized RNA sequencing gene expression data through GSEA software^27,28^ run using the hallmark MSigDB gene set collections. To examine the role of IH-ICC organoid in cell polarity and bile acid synthesis and metabolism, we cross-referenced the differential gene expression with the Gene Ontology (GO) annotations and predict the protein interactions networking using Search Tool for the Retrieval of Interacting Genes (STRING). Top canonical pathways analysis was performed using Ingenuity Pathway Analysis (IPA) (Qiagen).

### 2.16 GLuc HCVcc infection and analysis

GLuc HCVcc is a full length Gaussia luciferase reporter virus Jc1FLAG2 (p7-nsGluc2A)35 infectious Jc1 genome that allows us to monitor viral infection in real time. Knock down HCVcc is the corresponding polymerase-defective mutant that inhibits viral replication. Respective viral stocks were prepared using electroporation on Huh 7.5 cells as described [14]. Infectious titers of HCVcc inocula were determined using Huh 7.5 cells as described. Cultures were inoculated for 3 h with HCVcc then washed extensively with PBS and fed with culture media.

### 2.17 Cholyl-lysyl-fluorescein (CLF) transportation

5 μM CLF (Corning) was incubated with cultures treated with 10 μM Troglitazone (TGZ, Sigma) and 0.02% DMSO vehicle control for 45min. The cultures were then washed five times with PBS and incubated in culture media for 30min before confocal imaging. For CLF quantification, cultures where treated by cell lysis buffer (Sigma) and fluorescence intensity was measured using plate reader.

### 2.18 Animal experiments

All animal experiments were performed in accordance with UK Home Office regulations (UK Home Office Project License number PPL 70/8702). Immunodeficient NOD.Cg-Prkdc^scid^ Il2rg^tm1Wjl^/SzJ (NSG) mice which lack B, T and NK lymphocytes were bred in-house with food and water available ad libitum pre- and post-procedures. A mix of male and female animals were used, aged approximately 6–8 weeks. An incision was made in the capsule of the caudate lobe of the liver and a ‘pocket’ was created by raising a flap of capsule on either side of the incision. The scaffold was slid into the pocket and covered by the capsular flaps. The left lobe of the liver was then allowed to cover the site of the transplant.

### 2.19 Statistical analysis

N in the paper represents the number of biological replicates of each batch of liver specific differentiation performed using three iPSC lines. Statistical significance (p < 0.05) was determined by using Student's T-test (assume Gaussian distribution, two-tailed) or One-way ANOVA followed by Tukey's posthoc test. .

## 3 Results

### 3.1 iPSC-derived hepatic progenitors (IH) form organoids analogous to fetal liver derived primary cells following culture in ICC scaffolds

**Fig. 2:**
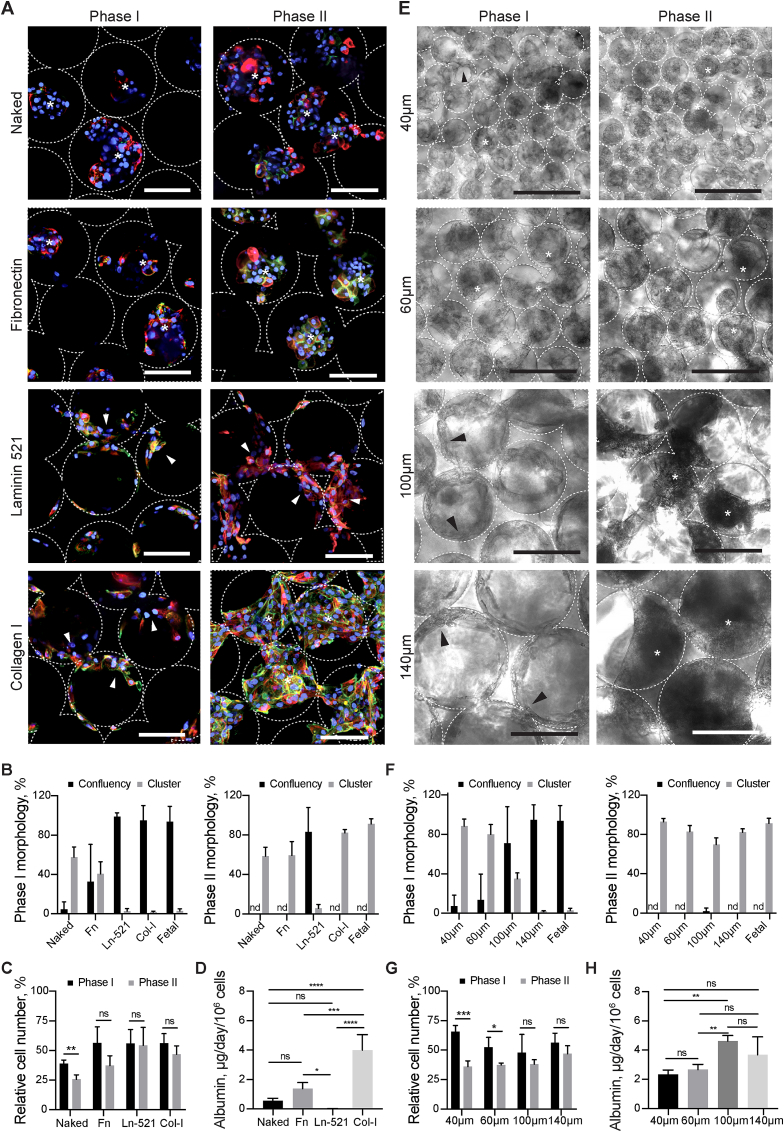
Characterizing the effects of pore size and ECM proteins on liver organoid formation in ICC scaffold. (**A**) Confocal micrographs of IH (CTNNB, green; CK19, red) following seeding into non-coated (Naked), Fibronectin (Fn), Laminin 521 (Ln-521), and Collagen I (Col-I) coated ICC's. (**B**) Quantification of cellular attachment (confluency) and cluster formation (cluster) ability seen across different ECM's during Phase I and II. (**C**) Cell number relative to initial cell seeding number in Phase I and II. (**D**) Albumin secretion rate of IH seeded in different ECM coated ICC in Phase II (**E**) Bright field images revealing the morphologies of IH in ICC with different collagen coated pore sizes (40 μm, 60 μm, 100 μm and 140 μm) and the respective (**F**) morphological quantitation and (**G**) relative cell number in Phase I and II. (**H**) Albumin secretion rate of IH seeded in ICC with different pore sizes in Phase II. Arrowheads indicate cells lining surface of ICC; asterisks represent cells forming clusters. Scale bar, 100 μm. Mean ± sd, N = 4. *p < 0.05; **p < 0.005; ***p < 0.0005; ****p < 0.0001; ns non-significant. (For interpretation of the references to colour in this figure legend, the reader is referred to the Web version of this article.)

Human fetal liver cells seeded into ICC scaffolds with the 140 μm pore size and type I collagen (Col-I) coating attach as a single cell layer (Phase I – days 0–5 post-seeding) before organising into morphologically stable, interconnected clusters (Phase II - days 7 post-seeding onward) (Fig. 1C). The resultant 3D structure resembles a mini-liver or hepatic ‘organoid’ with demonstrated advanced liver function [9]. We therefore hypothesized that organoid formation was critically dependent upon the translation of specific ‘cell-matrix’ and ‘cell-cell’ signals by hepatic progenitors in a manner which could be recapitulated using iPSC-derived progenitors (IH) (Supplementary Fig. 1). To test this hypothesis, we therefore engineered a library of ICC scaffolds to evaluate the effects of selected extracellular matrix protein (ECM) coatings and pore sizes on IH-organoid forming ability (Fig. 2). Of the ECM coatings tested, only cells seeded into Col-I were able to attach and self-organize into interconnected clusters throughout the scaffold as observed with primary fetal progenitors (Fetal) (Fig. 2A–C, Supplementary Fig. 2). Cells seeded into fibronectin (Fn) attached but failed to establish confluency whilst cells seeded into laminin-521 (Ln-521) attached and remained as such throughout the culture. The observed organoid forming ability was matched by functional parameters of liver tissue such as albumin secretion and hepatic gene expression (Fig. 2D and Supplementary Fig. 3). Having established the optimal ECM coating, we next went on to assess the effects of cell-cell interaction by testing type I collagen coated ICC's composed from different pore sizes. Accordingly, a 140 μm pore size was found to be optimal for organoid formation based on morphology and again confirmed by hepatic function and gene expression (Fig. 2E–H). Through this extensive series of experiments, we observed IH seeded into type I collagen coated ICC scaffolds of 140 μm pore size best replicated the results seen with primary fetal liver cells with minimal cell lost and death (Supplementary Figs. 4-5). Importantly, the two-phase process of organogenesis was observed only with IH and primary fetal cells but did not occur with primary adult or cancer-like hepatocytes (Huh 7.5 and HepG2) (Supplementary Fig. 6).

### 3.2 IH-ICC derived organoids demonstrate morphological and transcriptomic characteristics of human liver

**Fig. 3:**
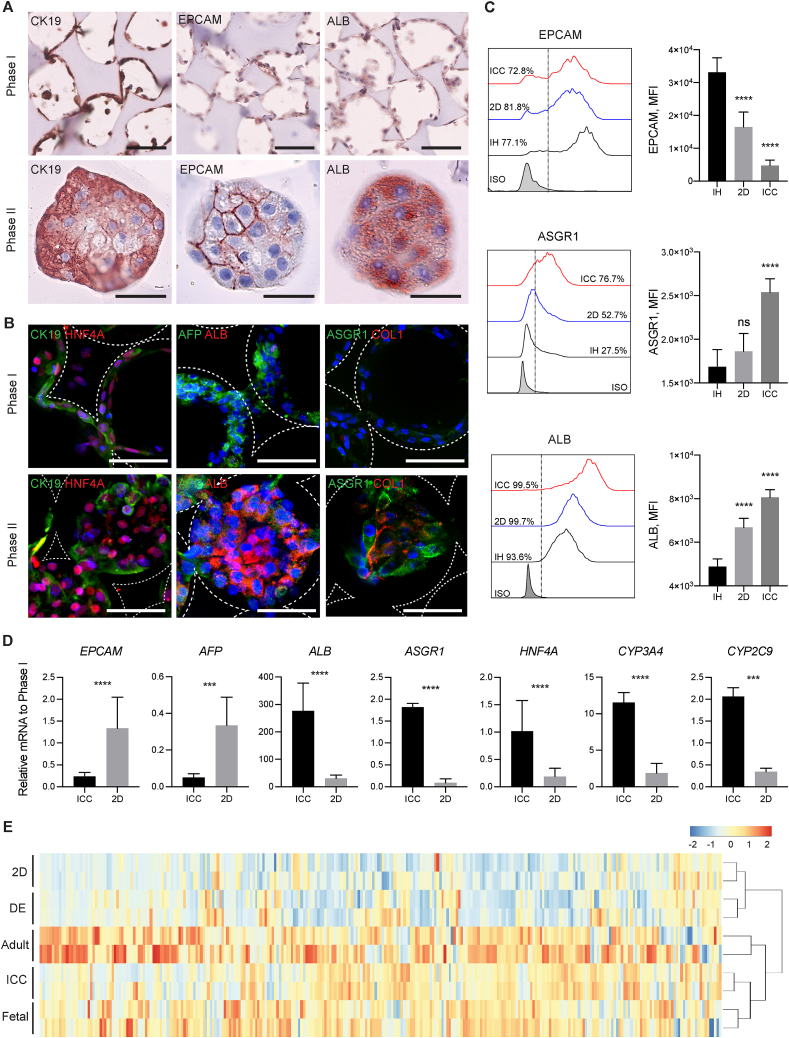
Morphological and transcriptomic characterization of IH-ICC organoids. (**A**) Histochemical images demonstrating morphogenesis of IH from a single cell layer (Phase I top panel; scale bar, 100 μm) to organoids (Phase II bottom panel; scale bar, 50 μm) occurs in conjunction with differential protein expression of developmental markers CK19 (left), EPCAM (middle) and ALB (right). (**B**) Confocal micrographs highlighting upregulated protein expression of mature (ALB, ASGPR1, COL1) hepatic markers occurs in conjunction with down regulation of immature (CK19 and AFP) markers during transition of IH from Phase I (top panel; scale bar, 100 μm) to Phase II (bottom panel; scale bar, 100 μm) organoids. (**C**) FACS histogram and mean fluorescence intensity (MFI) analysis demonstrating hepatic maturation kinetics of IH in ICC vs 2D culture (N = 4). (**D**) Differential gene expression (by RT-PCR) of selected genes reveals a more mature hepatic signature of IH in ICC vs. 2D culture (N = 8). (**e**) Bi-clustering heatmap of 296 liver-specific genes across different primary (adult & fetal liver) and IH (DE, 2D & ICC) samples. Samples are linked by the dendrogram above to show the similarity of their gene expression patterns. Mean ± sd, *p < 0.05; **p < 0.005; ***p < 0.0005; ****p < 0.0001; ns nonsignificant.

To evaluate the anatomical similarity between organoids derived from iPSC-hepatic progenitors cultured in ICC scaffolds (IH-ICC) and human liver, we first used a combination of histochemical and immunofluorescence staining to characterize the organoid tissue across the two phases of organogenesis previously described (Fig. 3A–B and Supplementary Figs. 7-10). In Phase I, hepatic progenitor markers such as keratin 19 (CK19), epithelial cell adhesion molecule (EPCAM) and alpha-fetoprotein (AFP) proteins were expressed in cells lining the circumference of the scaffold's pores. Following onset of organogenesis in Phase II, these (CK19, EPCAM, AFP, EdU) +ve progenitor cells were retained at the periphery of the organoid, with more mature cells, characterized by protein expression of asialoglycoprotein receptor 1 (ASGR1), albumin (ALB), and hepatocyte nuclear factor 4-alpha (HNF4A), forming and remodelled by the cell secreted type I collagen (Col-I) at the centre. These data were then validated quantitatively using flow cytometry (Fig. 3C) and by RT-PCR for gene expression (Fig. 3D), allowing us to conclude that organoids became established with unique cellular arrangement in a structure reminiscent of the hepatic buds seen in embryonic [15] and regenerating human liver tissue [16]. This anatomical distribution was not observed when IH were cultured in Matrigel, the current gold standard in the field, or the conventional 3D spheroid model. Embedding in Matrigel, IH established lumen-containing colonies, expressing mature hepatic markers (Supplementary Fig. 11). On the contrary, IH formed solid cell clusters in spheroid model with more mature cells segregated on the outer layer (Supplementary Fig. 12). Both Matrigel and spheroid models demonstrated functional properties (protein secretion and metabolic activity) significantly higher compared to 2D culture but notably not significantly higher than in ICC culture (Supplementary Fig. 13).

**Fig. 4:**
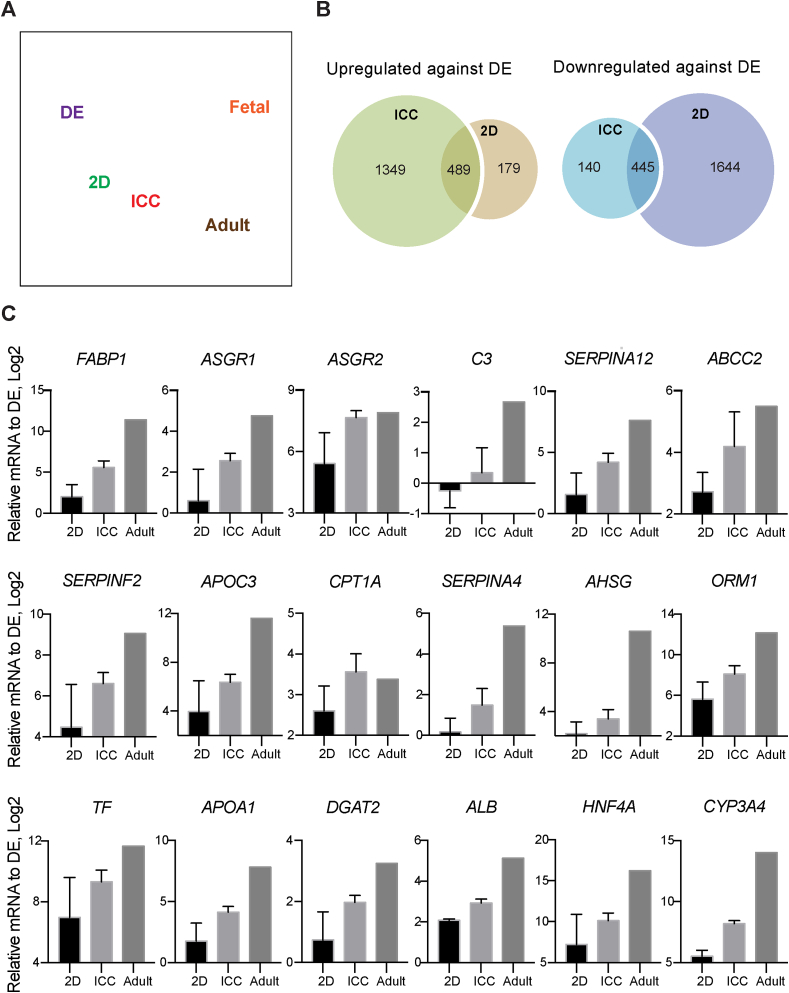
The transcriptomic analysis of liver organoid in ICC. (**A**) Principle component analysis of RNA-seq data demonstrating the proximity of gene expression variance in 2D plot. (**B**) Venn diagrams showing the number of up and downregulated gene in ICC and 2D with respect to DE. (**C**) RT-PCR validation on top 18 liver-specific genes identified by RNA-seq analysis (N = 3) Mean ± sd.

Having validated the organoid structure in our new bioengineered platform, we next sought to characterize the global transcriptomic changes during the different phases of organogenesis using RNA-seq. To do this, organoids were compared to iPSC derived definitive endoderm (DE), 2D cultured IH, primary adult and primary fetal hepatocytes. Principle component analysis (PCA) on DE, 2D, ICC, fetal and adult samples (Fig. 4A) shows that IH have a distinct transcriptional phenotype when cultured in ICC over 2D conditions. A liver-specific gene-set of 296 genes were selected to perform unbiased hierarchical clustering and visualized by heatmap. The unbiased hierarchical clustering revealed that the liver-specific transcriptional signature of organoids was positioned in the cluster next to adult and fetal liver, with higher similarity to fetal than adult, whereas 2D cells clustered next to DE (Fig. 3E). Overall 489 genes were found to be significantly upregulated in both organoid and 2D samples, with 1349 and 179 genes uniquely upregulated in organoid and 2D systems respectively (Fig. 4B). Gene set enrichment and Ingenuity Pathway (IPA) analysis of the upregulated genes revealed some liver-specific functions such as xenobiotic and bile acid metabolism to be enriched in both organoid and 2D platforms (Supplementary Fig. 14 and Supplementary Tables 1 and 2) but the majority of liver-specific functions such as cholesterol homeostasis, glycolysis, fatty acid metabolism and protein secretion were found to be uniquely enriched in IH-ICC. The top 18 upregulated organoid specific hepatic genes were validated by qPCR (Supplementary Table 3 and Fig. 4C).

Cumulatively, these data confirm our previous morphological observations suggesting IH derived progenitors seeded into ICC scaffolds form organoid structures whose transcriptomic and protein expression profiles resemble human liver structures.

### 3.3 IH-ICC organoids are functionally closer to human liver

**Fig. 5:**
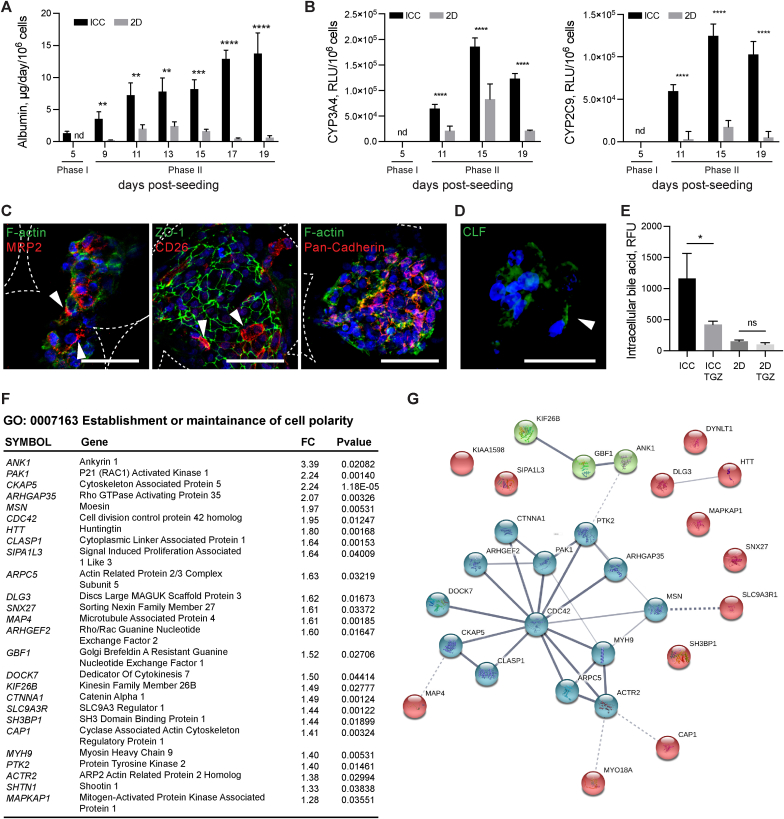
Functional validation of organoids. (**A**) Albumin secretion rate of IH-ICC vs. 2D (N = 8). (**B**) Basal metabolic activity of Cytochrome P450 isoforms CYP3A4 and CYP2C9 in IH-ICC vs 2D **(N** = **8**). RLU, relative luminescence unit. (**C**) Confocal micrographs showing expression of hepatocyte polarity markers (MRP2, ZO-1, CD26 and Pan-Cadherin) in IH-ICC organoids. Scale bar, 50 μm. White arrowhead points to apical region. (**D**) Accumulation of Cholyl-L-lysyl-fluorescein (CLS) in IH-ICC organoids after 40 min of CLF incubation followed by 40 min of washing. White arrowhead points to the CLF accumulation. (**E**) Effect of adding Troglitazone (TGZ) to CLS retention in IH-ICC (N = 4). (**F**) A list of uniquely upregulated genes in IH-ICC vs 2D that involved in establishment and maintenance of cell polarity. FC, fold change. (**G**) The STRING functional network predicted the associations between proteins (nodes) from regulated genes involved in cell polarity in IH-ICC. The cluster analysis was performed using KMEANS clustering algorithms. Mean ± sd, *p < 0.05; **p < 0.005; ***p < 0.0005; ****p < 0.0001; ns nonsignificant.

Upon extended interrogation, we discovered that IH-organoids are functionally more advanced than the 2D model in albumin production and basal activities of drug metabolizing cytochrome P450 isoforms CYP3A4 and CYP2C9 (Fig. 5A and B). Similarly, capability for transporting bile salts also appeared more advanced. Hepatic polarity proteins critical for the structure of bile canaliculi such as multidrug resistance protein 2 (MRP2), tight junction protein 1 (ZO-1), CD26 and pan-cadherin were all expressed (Fig. 5C and Supplementary Fig. 15). Bile salt efflux was then validated through uptake and accumulation of synthetic bile acid, cholyl-lysyl-fluorescence (CLF), in bile canaliculi-like regions (Fig. 5D). The ability of organoids to retain CLF was eliminated when treated with the BSEP inhibitor, Troglitazone (TGZ), as indicated by fluorescence measurement (Fig. 5E).

The cell polarity and bile acid secretory function of organoid was further validated by cross-referencing the uniquely upregulated differentially expressed genes in ICC with GO categories (GO 0007163: establishment or maintenance of cell polarity; GO 0000902: cell morphogenesis; GO 0007010: cytoskeleton organization; GO 0006699: bile acid biosynthetic process and GO 0008206: bile acid metabolic process) followed by functional network analysis using STRING on selected enriched gene sets (Fig. 5F and Supplementary Figs. 16-18) [17]. The 28 genes uniquely upregulated in organoids could be assigned to the key KEGG pathways such as regulation of actin cytoskeleton, focal adhesion and tight junction required for cell polarization (Fig. 5G).

### 3.4 IH-ICC organoids are suitable for disease modelling and form vascularised tissue following transplantation

**Fig. 6:**
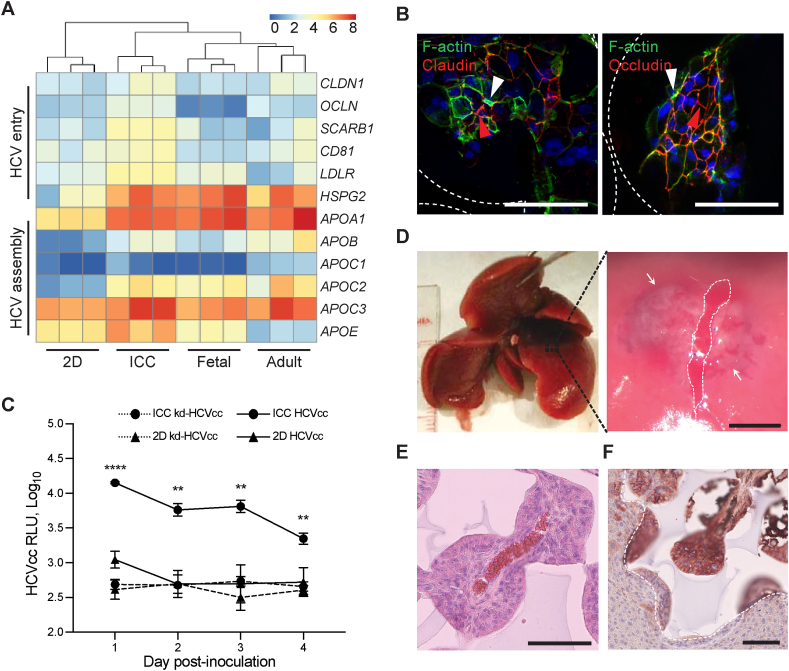
Disease modelling and in vivo transplantation. (**A**) Heatmap and hierarchal clustering comparing expression of 12 genes involved in encoding HCV entry and assembly in IH-ICC vs 2D vs primary (adult, fetal) liver. (**B**) Confocal imaging showing expression of claudin 1 and occludin in IH-ICC organoids. Scale bar, 100 μm. White and red arrowheads point to apical and lateral regions respectively. (**C**) HCV expression of IH-ICC vs 2D following infection with HCV reporter virus expressing secreted GLuc (HCVcc, N = 4) or mock infected with knock down HCVcc (kd-HCVcc, N = 3) and subsequently were sampled and washed daily. RLU, relative luminescence unit. (**D**) Photograph showing location of surgical pocket formation on murine left lateral lobe (left) and appearance following IH-ICC transplantation (right). The white dashed line depicts the capsular incision and the limits of the sub-capsular scaffold implant are shown by the white arrows. Scale bar 1.5 mm (**E**) H&E staining of explant reveals neo-vasculature of IH-ICC. Scale bar, 100 μm. (**F**) Immuno-histochemical staining of explant for human albumin. Dashed white line indicates the boundary between implant and host liver. Scale bar, 100 μm. Mean ± sd; **p < 0.005, ****p < 0.0001, nd not detected. (For interpretation of the references to colour in this figure legend, the reader is referred to the Web version of this article.)

Next, to test the ICC organoid's suitability for disease modelling we investigated whether organoids expressed HCV host factors and were susceptible to HCV infection. Transcriptional analysis revealed expression of HCV host factors were enriched in organoids and more closely resembled the expression signature of primary hepatocytes (Fig. 6A). This included genes responsible for HCV entry such as claudin 1 (*CLDN1*) occludin (*OCLN*) scavenger receptor, class B, type 1 (*SCARB1*), *CD81*, low-density lipoprotein receptor (*LDLR*) and heparin sulfate glycoprotein (*HSGP*). In addition, a wide range of apoliproteins responsible for packaging and interacting with viral particles were highly expressed [18]. Immunofluorescence staining showed claudin 1 and occludin appear to localize at the interface of cell-cell junctions, similar to ZO-1 and MRP2 (Fig. 6B and Supplementary Fig. 19). These results are in agreement with the claudin 1 distribution seen in HepG2 and Huh-7 cell models permissive to HCV entry [19,20]. We next infected organoids with genotype 2a HCV reporter virus expressing secreted *Gaussia* luciferase (HCVcc) and knock-down HCVcc (kd-HCVcc) which is incapable of replication and acts as a negative control. Luciferase signal was only detected in organoids inoculated with HCVcc cultures, whilst 2D cells and kd-HCVcc inoculated samples failed to exhibit detectable signal (Fig. 6C).

Having confirmed the organoid's preferential suitability for drug metabolism and disease modelling we next sought to explore the effects of in vivo transplantation. A pocket on the caudate lobe of murine liver was created by making an incision in the liver capsule. Organoids were placed into this pocket and sandwiched in place between the left lobe and the lower lateral lobe in order to achieve a bona fide homeostatic environment (Fig. 6D). After 4 weeks, grafts were retrieved for further analysis. H&E staining revealed implants were well integrated into the host parenchyma, without evidence of significant fibrosis/inflammation whilst neo vascularization had successfully occurred between host and donor tissues (Fig. 6E and Supplementary Fig. 20A–B). Histochemical staining with human albumin confirmed the implanted structures were of human origin, the organoid structure had remained intact and the presence of human albumin in host serum suggested cells remained functional (Fig. 6F and Supplementary Fig. 20C–E).

### 3.5 TGFβ and hedgehog signalling pathways are important for organoid formation

**Fig. 7:**
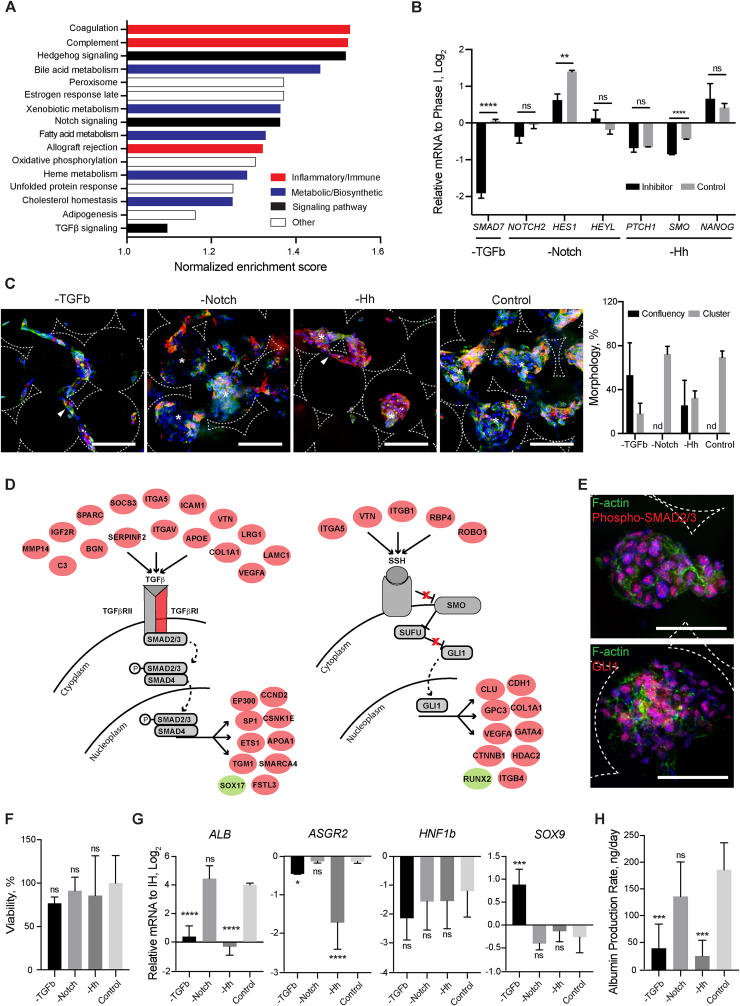
Mechanisms of organoid formation. (**A**) Bar chart detailing gene sets uniquely enriched in IH-ICC over 2D. (**B**) Gene expression (by RT-PCR) in IH-ICC of selected pathway transcriptional targets, TGFb, Notch and Hedgehog (Hh) following addition of respective inhibitors (N = 4). (**C**) Confocal micrographs of IH in Phase II (CTNNB, green; CK19, red) demonstrating effects of inhibitors on IH-ICC morphogenesis. Morphological quantification of observations provided on right. (**D**) Regulatory networks of TGFb (left) and hedgehog (right). Genes labelled in red and green were identified as significantly up- and down regulated in IH-ICC over 2D. (**E**) Confocal micrographs showing translocation of phosphor-SMAD2/3 and GLI1 in response to TGFb and hedgehog pathway activation in IH-ICC organoids. (**F**) Cell viability in IH-ICC as a consequence of adding each inhibitor as determined by cell activity (N = 8). (**G**) Gene expression (by RT-PCR) of selected hepatic and biliary markers in IH-ICC following addition of each inhibitor above (N = 4). (**H**) Effect of inhibitors on IH-ICC hepatic function by albumin production rate (N = 4). Mean ± sd, *p < 0.05, **p < 0.005, ***p < 0.0005, ****p < 0.0001, ns nonsignificant. (For interpretation of the references to colour in this figure legend, the reader is referred to the Web version of this article.)

To identify signalling pathways involved in the orchestration of hepatic organoid formation, gene set enrichment analysis was performed as described before. The top 15 gene sets uniquely enriched in the ICC were related to metabolic/biosynthetic and inflammatory/immune related processes (Fig. 7A). The enrichment of bile acid metabolism, xenobiotic metabolism, fatty acid metabolism, heme metabolism and cholesterol homeostasis are encouraging signs of liver-specific organogenesis. Notably, three highly conserved developmental pathways were identified through this analysis – hedgehog, notch and TGFβ. To confirm their functional relevance, we treated organoids with small molecule inhibitors of hedgehog (Cyclopamine – CYC, 0.2 μM), notch (DAPT, 10 μM) and TGFβR-1 (RepSox, 12.5 μM) and characterized the resultant effects on organoid formation. Morphological observations were also correlated with RT-qPCR evaluation of direct transcriptional targets for each signalling pathway (Fig. 7B). Cells managed to establish Phase I morphology (where cells lined up the surface of ICC) regardless of the treatment. However, cells treated with RepSox and CYC were unable to form typical organoid structures (Phase II), whilst DAPT treatment appeared to have little effect (Fig. 7C). RepSox treated cells arrested in Phase I of organogenesis resembling the observations seen with adult hepatocyte and liver carcinoma cells (Supplementary Fig. 6). CYC treated cells on the other hand, instead of transitioning into typical organoid structures, formed much smaller clusters that were less uniform in size and with a rougher surface. The regulatory network analysis on TGFB and hedgehog signalling pathways revealed several upstream ligands that are significantly up regulated in IH-ICC over 2D and could potentially serve as the ligand for signalling activation (Fig. 7D). The activation of these two pathways in IH-ICC were further validated by the translocation of phopho-SMAD2/3 and zinc finger GLI1 as shown in immunofluorescence staining (Fig. 7E and Supplementary Fig. 21). To ensure cytotoxicity was not the causal effect, the selected concentration of each inhibitor used was validated to have had minimal to no significant impact on cell viability (Fig. 7F). Furthermore, the dramatic change in organogenesis observed corresponded to a direct pathway effect (Fig. 7G), reduced hepatic gene expression profile (Fig. 7F), and reduced albumin production rate (Fig. 7H).

## 4 Discussion

Human organs exist in an incredible variety of shapes and sizes. Understanding the processes through which these varied forms arise during development, are maintained in homeostasis or become perturbed during disease represent some of the most fundamental questions facing us today in biology. iPSC-derived cells provide an excellent model with which to study such questions through ex vivo formation of mini organs known as organoids. iPSC-derived organoids are also highly appealing because they themselves could be used for patient specific disease modelling and therapy. Here we report a new approach to the generation of iPSC-derived hepatic organoids, using state of the art bio-engineering technology that by-passes the need for Matrigel and supporting cell co-culture. Utility of this platform was demonstrated by our findings in organoids of advanced hepatic function and dependence on key developmental signalling pathways (Hedgehog and TGFβ).

Ex vivo liver organogenesis is particularly challenging due to the daunting complexity of the organ's structure and function. A pivotal breakthrough in this field was made several years ago by Takebe and colleagues when they generated hepatic organoids by co-culturing iPSC-derived hepatic endoderm, HUVECs and MSCs in Matrigel [1]. Immediate downstream translational applications are potentially limited however by dependence upon non FDA-compliant materials such as mouse-sarcoma-derived Matrigel and the difficulty of scaling up production when using multiple cell types (MSCs & HUVECs) [6]. A more recent and equally exciting organoid generation system, developed by Huch & Clevers [2], is similarly limited by the need for Matrigel but also by its need for primary tissue. In this study, we therefore sought to engineer liver organoids free from Matrigel, MSCs, and HUVECs, using a 3D synthetic hydrogel scaffold and hiPSC-derived hepatic progenitors alone. To that end we developed a 3D scaffold called ICC (inverted colloidal crystal) resembling the precise architectural microenvironment into which the liver diverticulum engages during liver bud formation [15]. Due to the ICC's unique bottom-up fabrication approach, we were able to custom engineer ‘cell-matrix’ interactions through coating the inner lining of the ICC pores with select ECM proteins [15] as well as custom engineer ‘cell-cell’ interactions through manipulating pore size [8]. These engineering designs cumulatively sought to recapitulate the extracellular niche sensed by hepatic progenitors during bud formation. We observed that the initial bud morphology, which we describe in this study as Phase I organogenesis/budding, could only be achieved when two critical factors were met. First, presence of a suitable ECM coating to facilitate cell attachment which otherwise fails due to the bioinert property of the PEG from which the ICC is manufactured (Fig. 2A–D). Second, seeding into a suitably sized pore, which otherwise forces cells to clump rather than attach to the scaffold's architecture if pore sizes are too small (Fig. 2E–H). Importantly, cell condensation (as opposed to cell-ECM engagement) was associated with down-regulation in hepatic gene expression, lower albumin production rates and failure to progress to Phase II in which cells lining the ICC self-organize into interconnected clusters to create a homogenously repeating organoid tissue structure. This series of morphogenic movements (Phase I → Phase II) was unique to iPSC (IH) and primary (fetal) hepatic progenitors and did not occur with terminally differentiated adult cells or with cancer cell lines (Supplementary Fig. 6) [21,22]. We therefore hypothesize our 3D scaffolds leverage the intrinsic ability of liver stem/progenitor cells to form organized structures in a manner which conserves the interaction observed between progenitors and their surrounding niche during organogenesis/budding [23].

As a consequence of this two-phase organogenesis/budding, the ICC engineered organoids produce structures with progenitor cells at the periphery and more mature cells in the middle. Such arrangement is consistent with descriptions of early mammalian liver development, where liver diverticulum progressing from single cell lining to pseudostratified epithelium then three-dimensional liver bud [25]. This bioengineering approach may therefore provide a useful, but simplistic model to further our understanding of early liver development (budding) in vivo (Fig. 3A and B). This configuration is in direct contrast to findings seen with spheroid/cell condensation culture systems where cells at the periphery are more viable and proliferative than cells at the core due to hypoxia and DNA damage [[26], [27], [28], [29]]. That difference underscores the success of the engineering feat achieved here. The fact that liver specific drug metabolism and disease modelling become upregulated in parallel with these morphogenic changes along with the capacity of the organoids to integrate, neo-vascularise and survive in vivo emphasizes the physiological relevance of the tissue created here (Fig. 4, Fig. 5). How such morphogenesis and zonation is programmed has been a long-standing question in biology. Our initial interrogation suggests both the TGFβ and hedgehog signalling pathways to be pivotal in this regard. Hedgehog signalling is particularly interesting in the context of liver zonation because it has been reported to orchestrate the position of hepatocytes along an oxygen gradient [24]. Our treatment of progenitors with a hedgehog signalling pathway inhibitor, Cyclopamine, generated not only smaller but dysmorphic organoids (Fig. 6C and D) and was accompanied by downregulation of liver function (Fig. 6F and G). These data suggest Hedgehog may be involved in much more than simple cell positioning therefore.

To our knowledge, this is the first attempt to produce liver organoids using just the combination of iPSC-derived hepatic progenitors and a synthetic hydrogel. The unique modular features of the ICC scaffold will allow study of the complex, combinatorial influences of physical and chemical signals during liver organogenesis in a physiologically relevant, dissectible 3D microenvironment. In addition, the scalable structure and clinically compliant material (PEG) and cell lines [11] used, opens up the possibility of future human therapy. These results highlight the enormous potential of bioengineered organoids for discovery and translational science.

## Data availability statement

The raw/processed data required to reproduce these findings cannot be shared at this time due to technical or time limitations.

## Disclosures

The authors declare no conflicts of interest relevant to the study presented here.

## Author contributions

All authors contributed extensively to the work presented in this paper. S.S.N., C.W.F, N.J.C., H.N., J.S.G. and S.T.R. developed the study concept and design. S.S.N., K.S., J.M.S., M. P. S., S.J.I.B., M.H.L. and D.Y.N. acquired data. S.S.N., K.S., J.M.S., J.S.G. and S.T.R. analyzed and interpreted the data. S.S.N., J.S.G. and S.T.R. drafted the manuscript and revised the manuscript for important intellectual content. J.S.G. and S.T.R. supervised the study.

## Grant support

National Institute for Health Research (NIHR), United Kingdom; Guy's and St Thomas' NHS Foundation Trust, United Kingdom; NIHR Biomedical Research, United Kingdom; Medical Research Council (MRC) (MGSBACR), United Kingdom; Alpha-1 Antitrypsin Foundation, United Kingdom; National Institutes of Health (R01AI099245 and U19AI109662), United State of America; Burroughs Wellcome Fund Clinical Scientist Award in Translational Research; California Institute for Regenerative Medicine (CIRM) (LA1_C12-06917); National Research Foundation of Singapore through a Competitive Research Programme grant (NRF-CRP10-2012-07).

## References

[1] Takebe T. (2013). Vascularized and functional human liver from an iPSC-derived organ bud transplant. Nature.

[2] Huch M. (2013). In vitro expansion of single Lgr5+ liver stem cells induced by Wnt-driven regeneration. Nature.

[3] Sato T. (2009). Single Lgr5 stem cells build crypt–villus structures in vitro without a mesenchymal niche. Nature.

[4] Lancaster M.A. (2013). Cerebral organoids model human brain development and microcephaly. Nature.

[5] Takasato M. (2015). Kidney organoids from human iPS cells contain multiple lineages and model human nephrogenesis. Nature.

[6] Fatehullah A., Tan S.H., Barker N. (2016). Organoids as an in vitro model of human development and disease. Nat. Cell Biol..

[7] Gjorevski N. (2016). Designer matrices for intestinal stem cell and organoid culture. Nature.

[8] Shirahama H. (2016). Fabrication of inverted colloidal crystal poly(ethylene glycol) scaffold: a three-dimensional cell culture platform for liver tissue engineering. J. Vis. Exp..

[9] Ng S.S. (2017). Long-term culture of human liver tissue with advanced hepatic functions. JCI Insight.

[10] Rashid S.T. (2010). Modeling inherited metabolic disorders of the liver using human induced pluripotent stem cells. J. Clin. Invest..

[11] Baghbaderani B.A. (2015). Cgmp-manufactured human induced pluripotent stem cells are Available for pre-clinical and clinical applications. Stem Cell Rep..

[12] Gramignoli R. (2012). Development and application of purified tissue dissociation enzyme mixtures for human hepatocyte isolation. Cell Transplant..

[13] Hannan N.R.F. (2013). Production of hepatocyte-like cells from human pluripotent stem cells. Nat. Protoc..

[14] Lussignol M. (2016). Proteomics of HCV virions reveals an essential role for the nucleoporin Nup98 in virus morphogenesis. Proc. Natl. Acad. Sci. U. S. A..

[15] Gordillo M., Evans T., Gouon-Evans V. (2015). Orchestrating liver development. Development (Cambridge, England).

[16] Stueck A.E., Wanless I.R. (2015). Hepatocyte buds derived from progenitor cells repopulate regions of parenchymal extinction in human cirrhosis. Hepatology.

[17] Hua M. (2012). Molecular mechanisms regulating the establishment of hepatocyte polarity during human hepatic progenitor cell differentiation into a functional hepatocyte-like phenotype. J. Cell Sci..

[18] Lee J.Y. (2014). Apolipoprotein E likely contributes to a maturation step of infectious hepatitis C virus particles and interacts with viral envelope glycoproteins. J. Virol..

[19] Mee C.J. (2009). Polarization restricts hepatitis C virus entry into HepG2 hepatoma cells. J. Virol..

[20] Molina-Jimenez F. (2012). Matrigel-embedded 3D culture of Huh-7 cells as a hepatocyte-like polarized system to study hepatitis C virus cycle. Virology.

[21] Kim M.H. (2016). Biofunctionalized hydrogel microscaffolds promote 3D hepatic sheet morphology. Macromol. Biosci..

[22] Kim M.H. (2015). Phenotypic regulation of liver cells in a biofunctionalized three-dimensional hydrogel platform. Integr. Biol..

[23] Wang Y. (2016). ECM proteins in a microporous scaffold influence hepatocyte morphology, function, and gene expression. Sci. Rep..

[24] Kietzmann T. (2017). Metabolic zonation of the liver: the oxygen gradient revisited. Redox Biol..

[25] Turner R. (2011). Human hepatic stem cell and maturational liver lineage biology. Hepatology (Baltimore, Md.).

[26] Riffle S. (2017). Linking hypoxia, DNA damage and proliferation in multicellular tumor spheroids. BMC Canc..

[27] Bell C.C. (2016). Characterization of primary human hepatocyte spheroids as a model system for drug-induced liver injury, liver function and disease. Sci. Rep..

[28] Gaskell H. (2016). Characterization of a functional C3A liver spheroid model. Toxicol. Res..

[29] Langan L.M. (2016). Direct measurements of oxygen gradients in spheroid culture system using electron parametric resonance oximetry. PLoS One.

