# Supplementary material for: Human iPS derived progenitors bioengineered into liver organoids using an inverted colloidal crystal poly (ethylene glycol) scaffold
Source: Biomaterials. 2018 Nov;182:299–311. doi: 10.1016/j.biomaterials.2018.07.043 (PMC6131727; doi:10.1016/j.biomaterials.2018.07.043)
Supplement: Multimedia component 1 [file mmc1.docx]

**Supporting Data
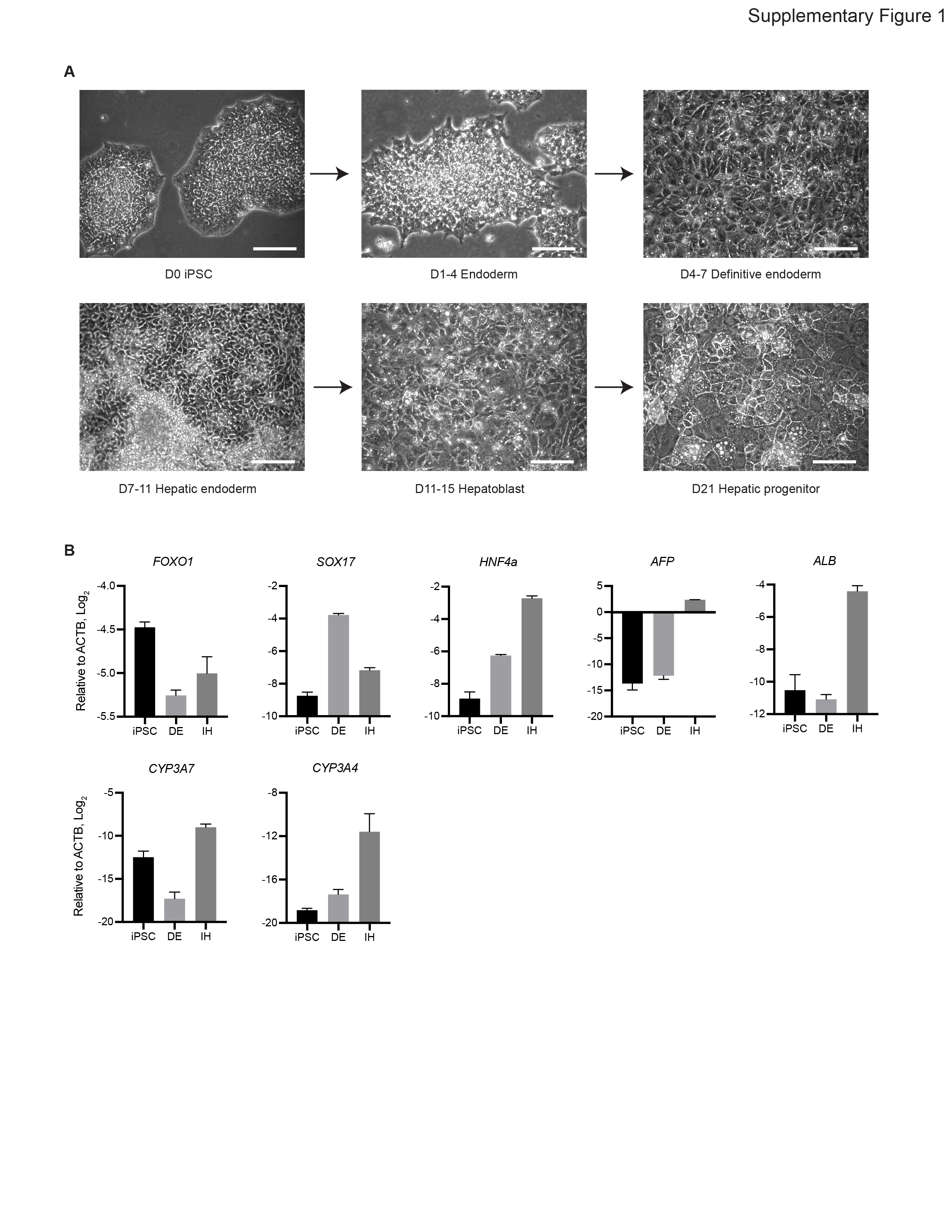
Supplementary Fig. 1.** The differentiation of iPSC-derived hepatic progenitors. (**A**) Brightfield images of cells at different stages of differentiation. Scale bar, 100µm. (**B**) Differential gene expression using RT-PCR revealing a direct liver differentiation from iPSCs to IH. Mean±sd, N=4.


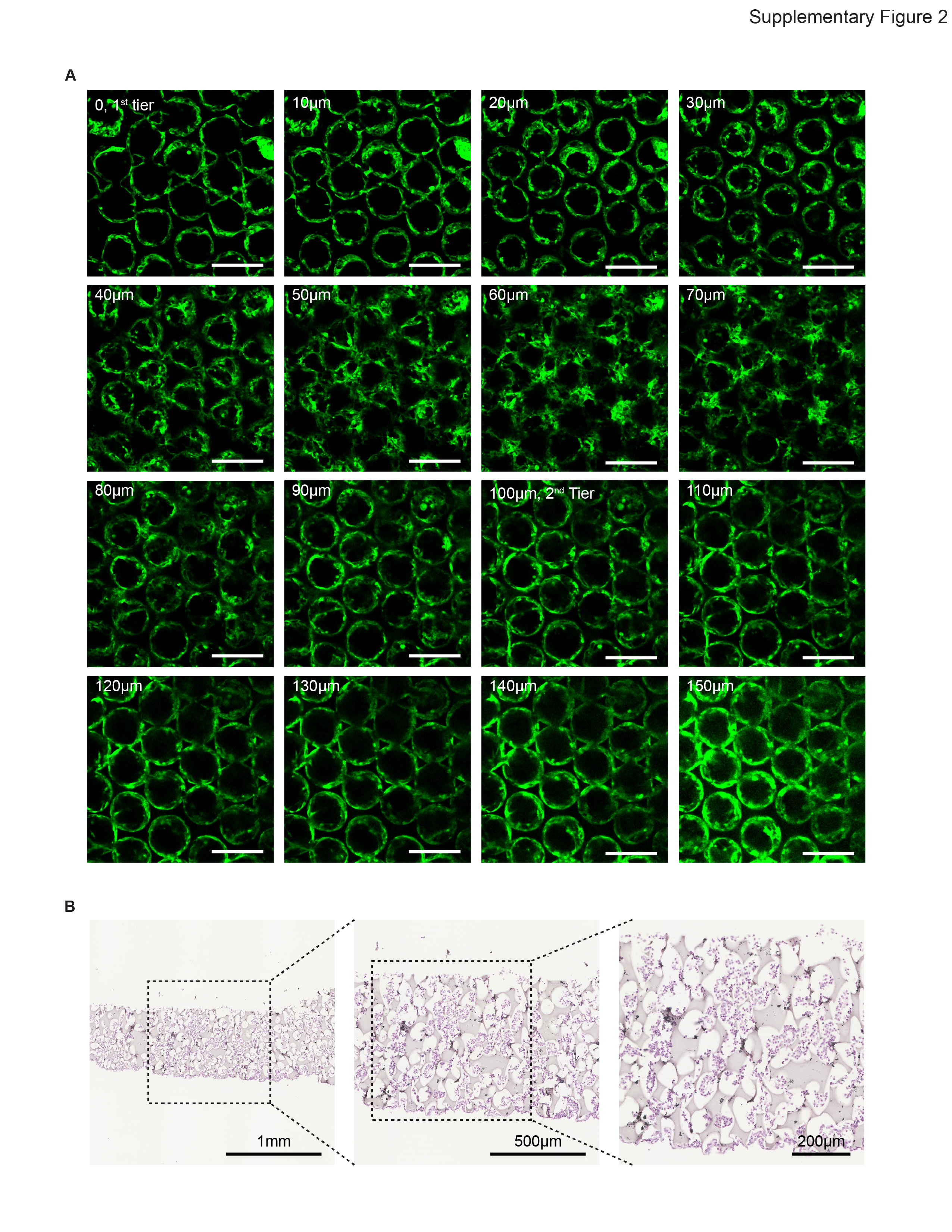
 **Supplementary Fig. 2.** Confocal and histochemical images demonstrating uniform cell distribution across the z-axis of scaffold. (**A**) A series of confocal Z-stack micrographs showing F-actin positive IH from first to second tier of ICC scaffold with 10µm interval. Scale bar, 100µm. (**B**) H&E images demonstrating uniform distribution of interconnected cell clusters across ICC.

**
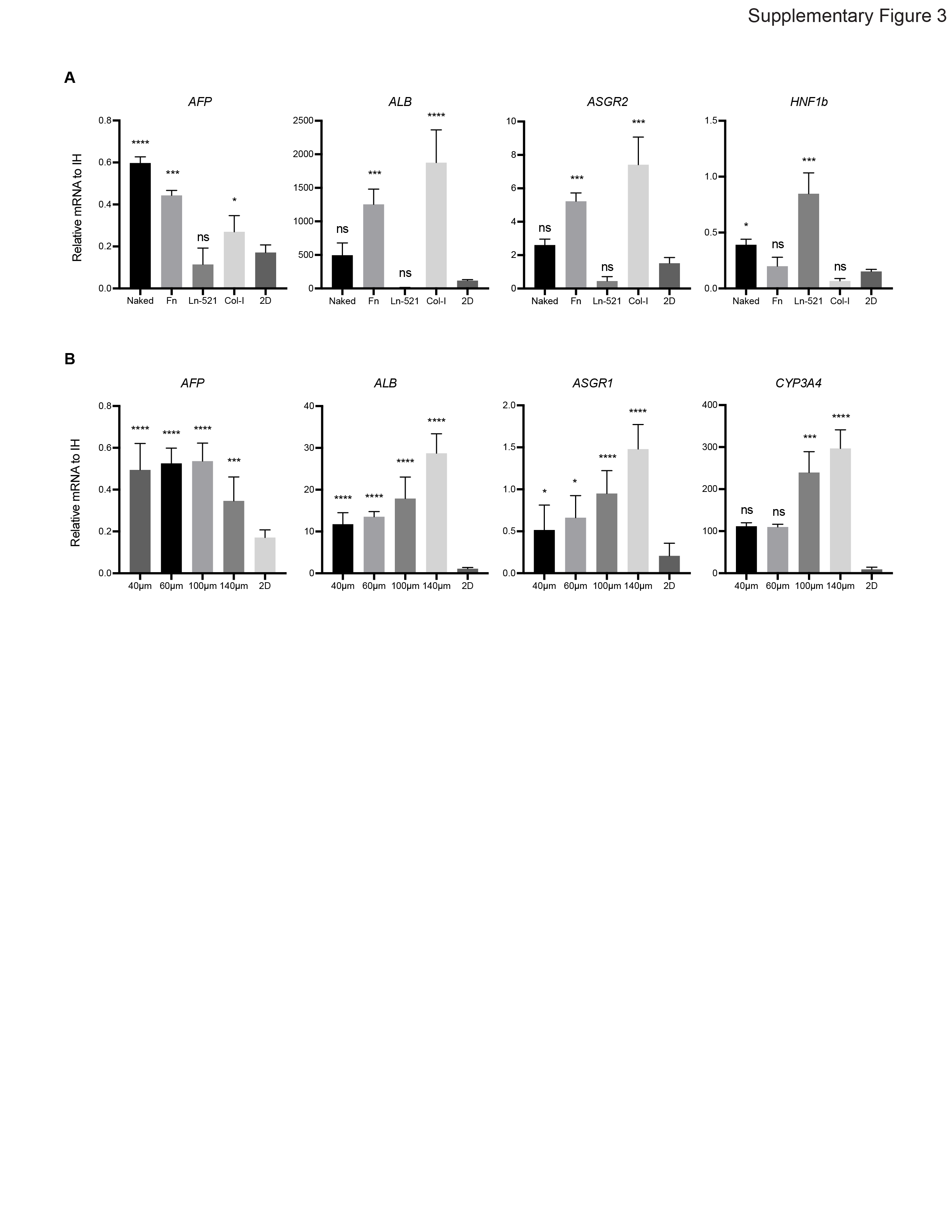
Supplementary Fig. 3.** Differential gene expressions of four hepatic markers on ICC with four different ECM proteins and pore sizes with respect to 2D counterpart. Mean±sd, N=8. *p< 0.05; **p< 0.005; ***p< 0.0005; ****p<0.0001; ns non-significant; nd not detected.

**
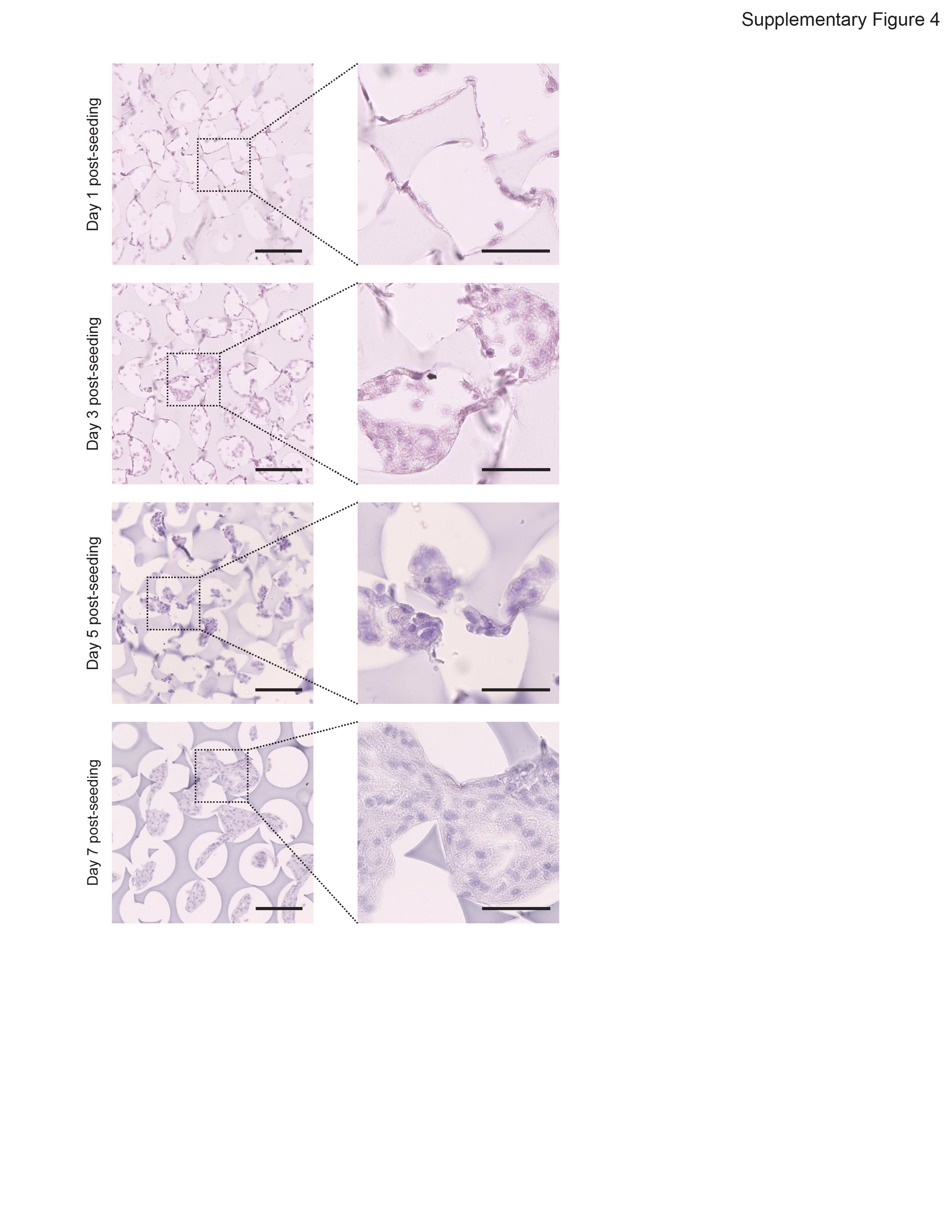
**

**Supplementary Fig. 4.** Time course of IH-organoid formation in type I collagen coated ICC with 140µm pore size demonstrated by H&E staining. Left panel, scale bar, 200µm. Right panel, scale bar, 100µm.


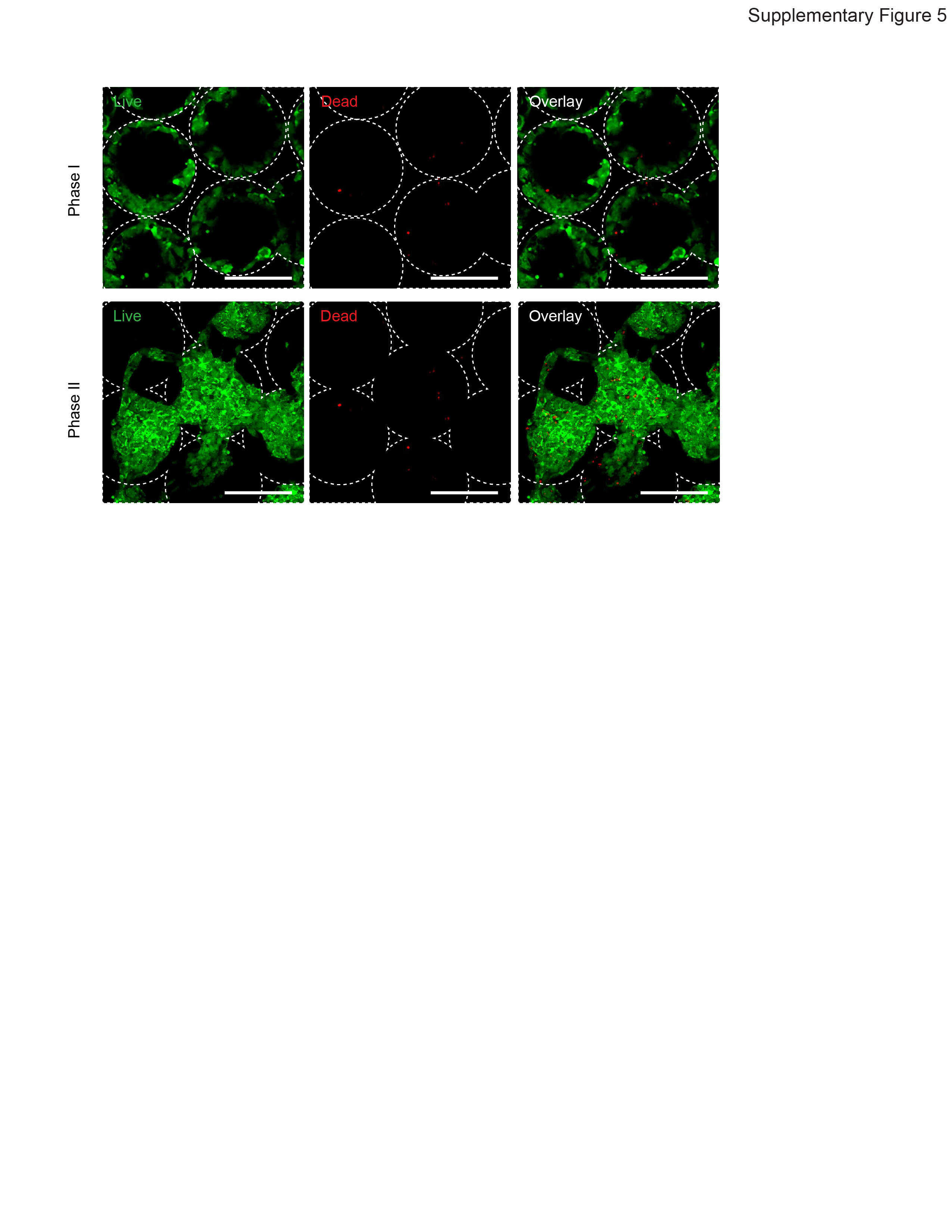
**Supplementary Fig. 5.** Live/ dead confocal micrographs of IH in type I collagen coated ICC with 140µm pore size. Minimal cell death was detected in both phases Scale bar, 100µm.

**
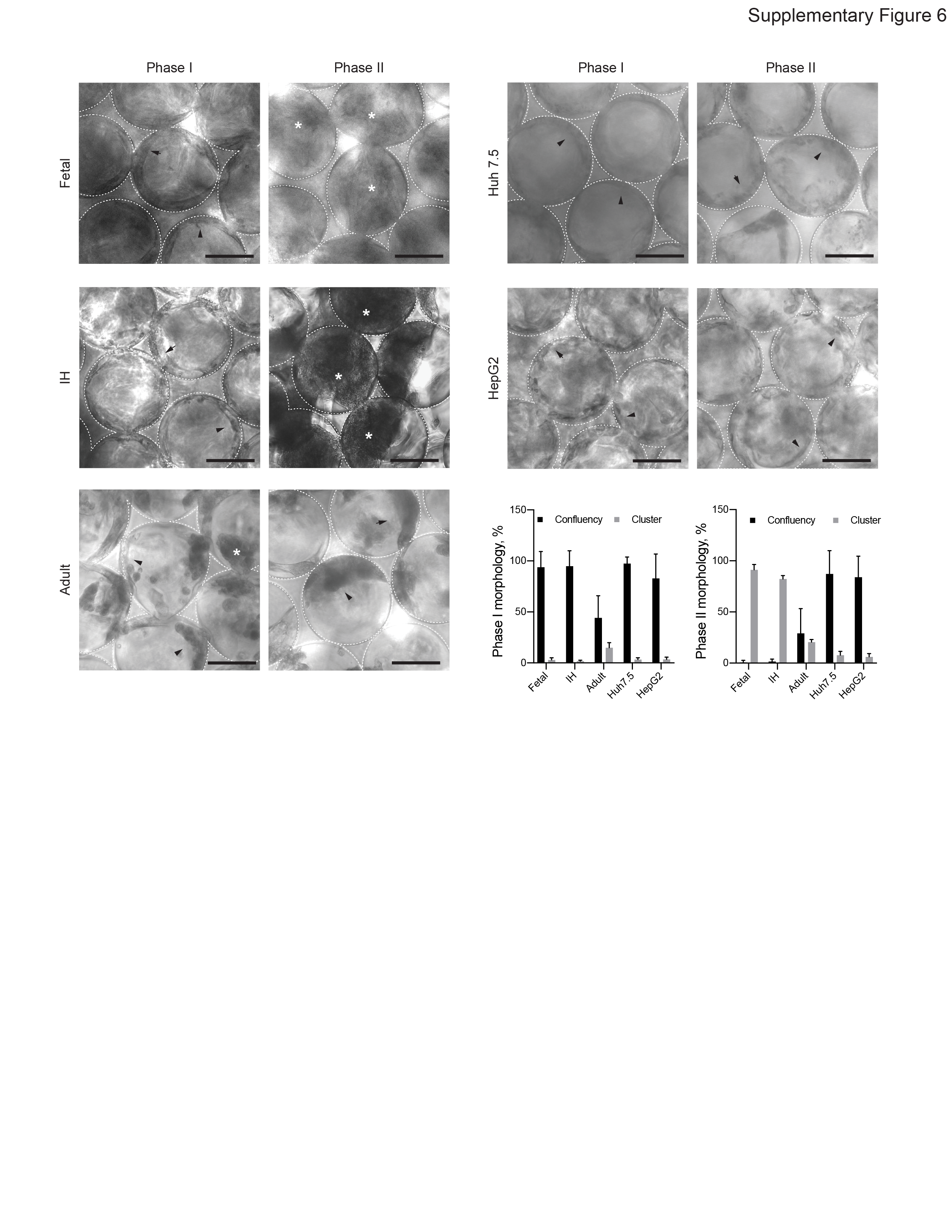
Supplementary Fig. 5.** Bright field images of primary fetal liver cells (Fetal), IH, human adult primary hepatocytes (Adult), Huh 7.5 and HepG2 cells following seeding into collagen coated ICC’s. Morphological quantification of observations provided on right. Arrowheads indicate cells lining surface of ICC; asterisks represent cells forming clusters. Scale bar, 100µm**.**


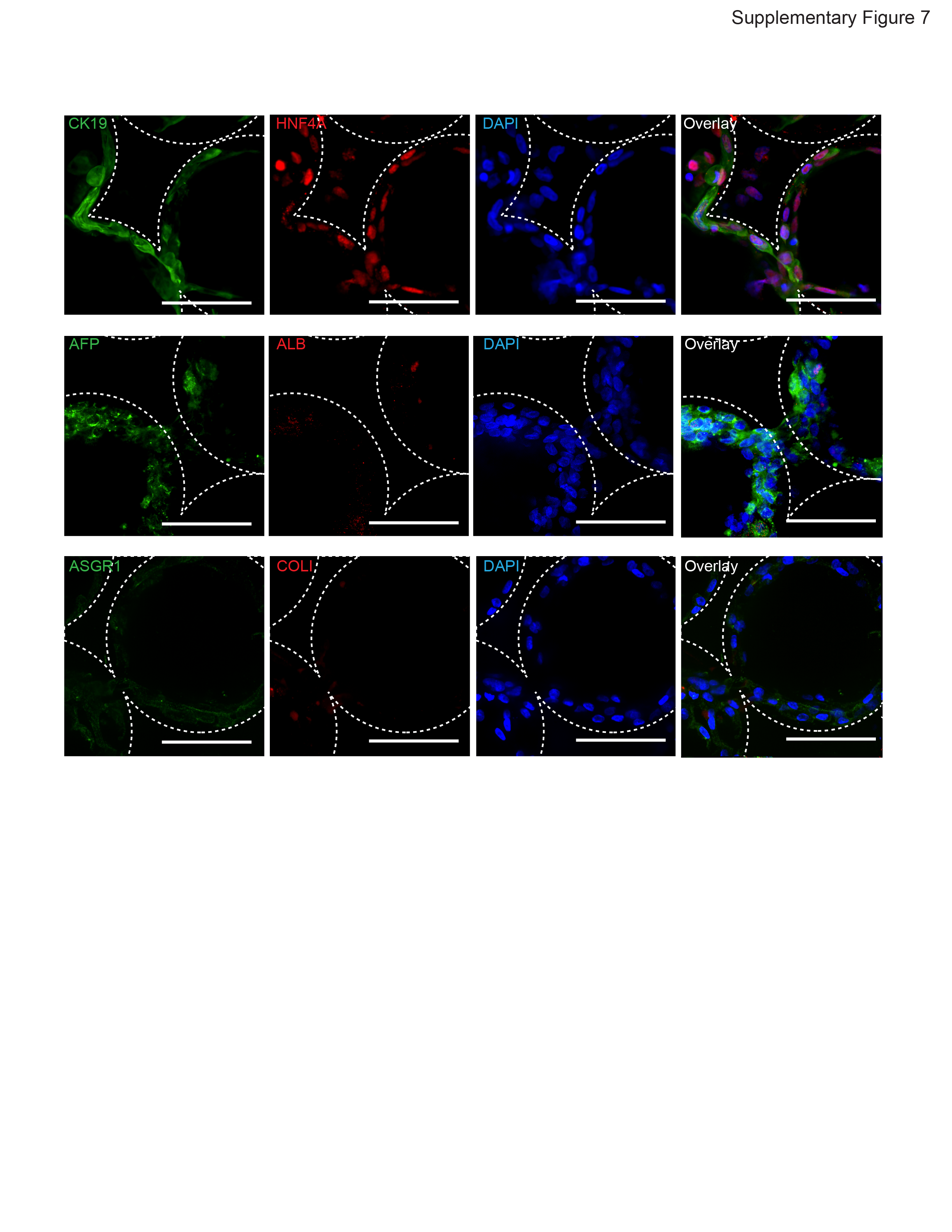
**Supplementary Fig. 7.** Single channel and overlay confocal micrographs of images shown in top panel of Fig. 3B. Scale bar, 100µm.

**
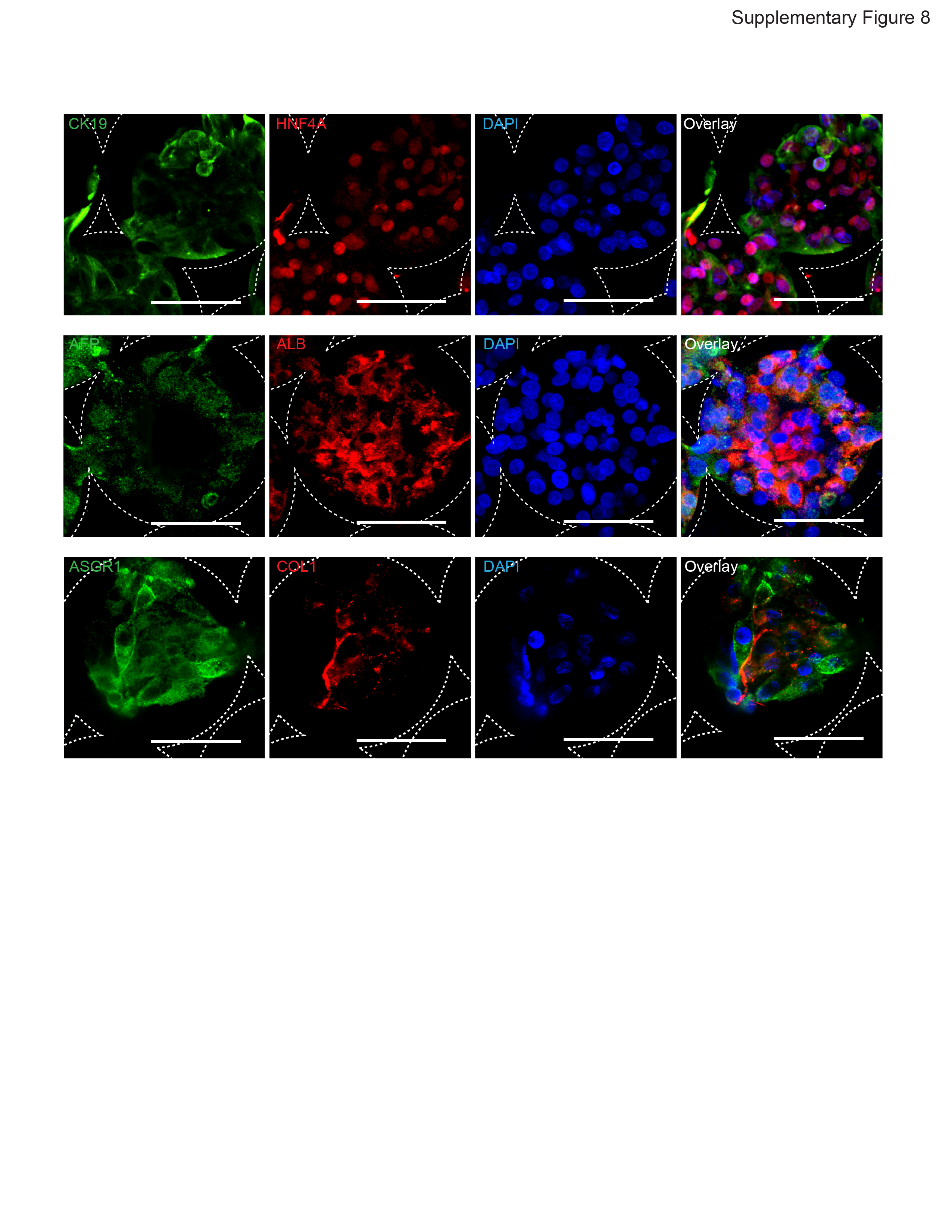
Supplementary Fig. 8.** Single channel and overlay confocal micrographs of images shown in bottom panel of Fig. 3B. Scale bar, 100µm.


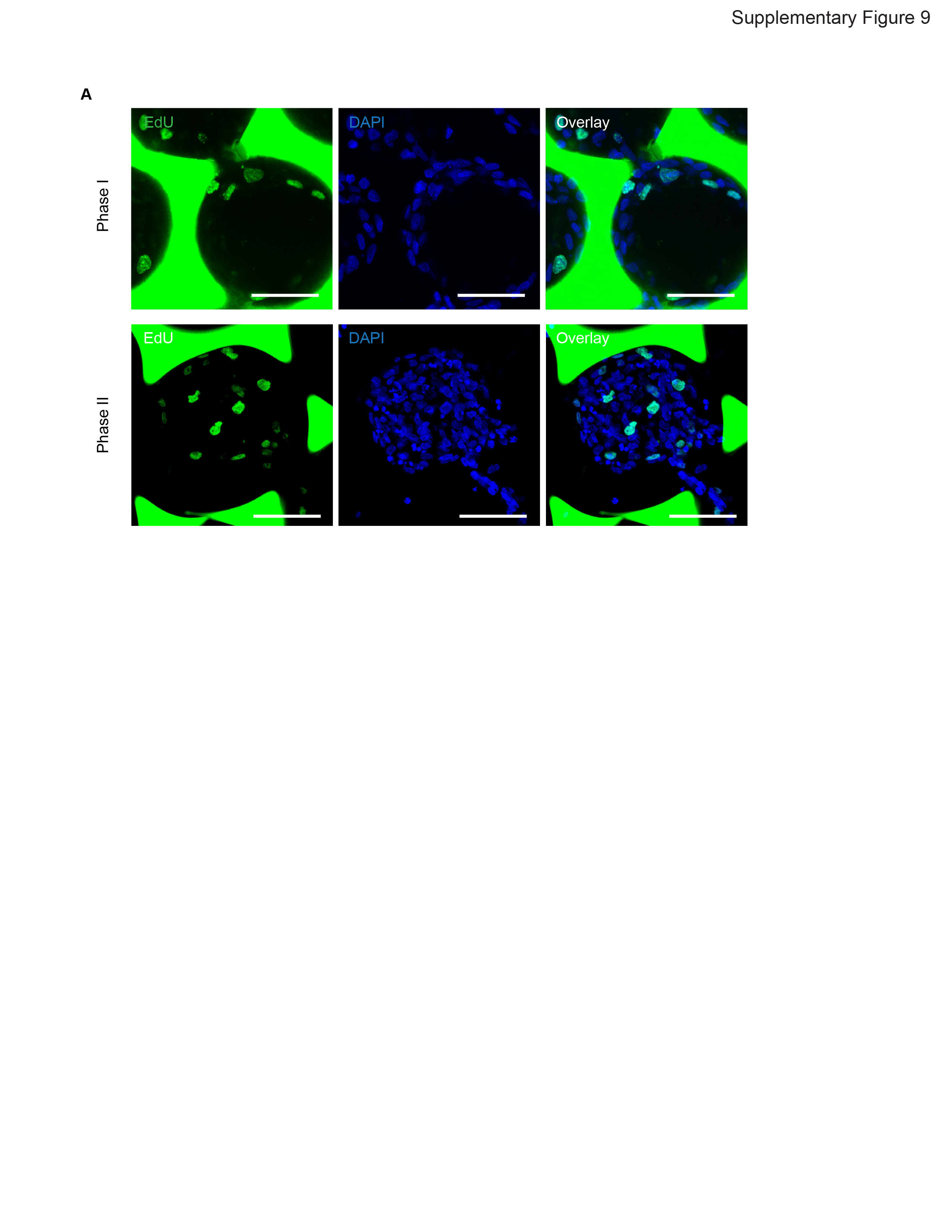
**Supplementary Fig. 9.** Confocal micrographs revealing proliferative cells in IH-ICC. EdU positive cells are uniquely expressed on the peripheral of cell clusters at Phase II. Scale bar, 50µm.

**
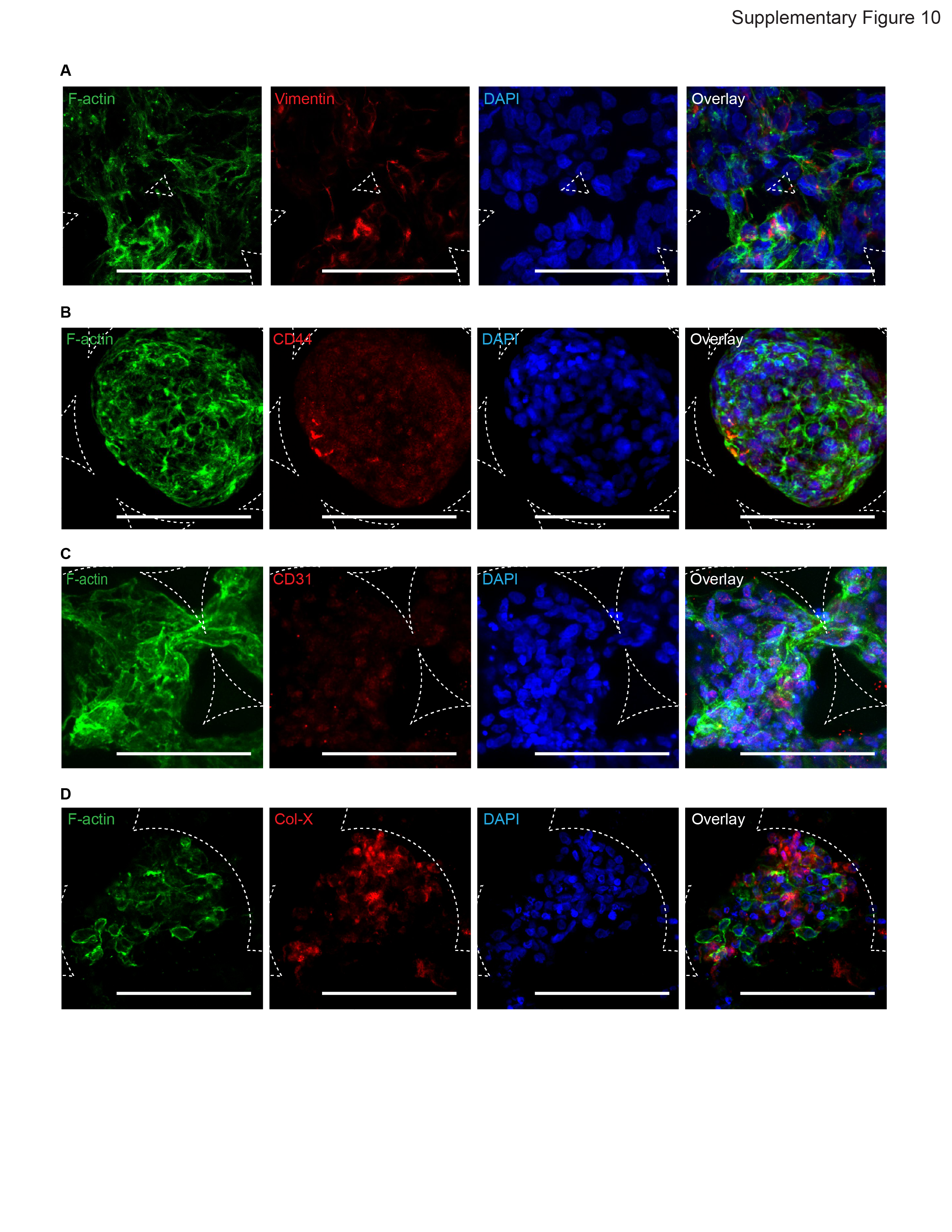
Supplementary Fig. 10.** Confocal micrographs showing liver nonparenchymal cells such as (**A**) fibroblasts (Vimentin positive cells), (**B**) mesenchymal cells (CD44 positive cells) and (**C**) endothelial cells (CD31 positive cells) and deposition of (**D**) type X Collagen (Col-X) in IH-ICC at Phase II. Scale bar, 100µm.


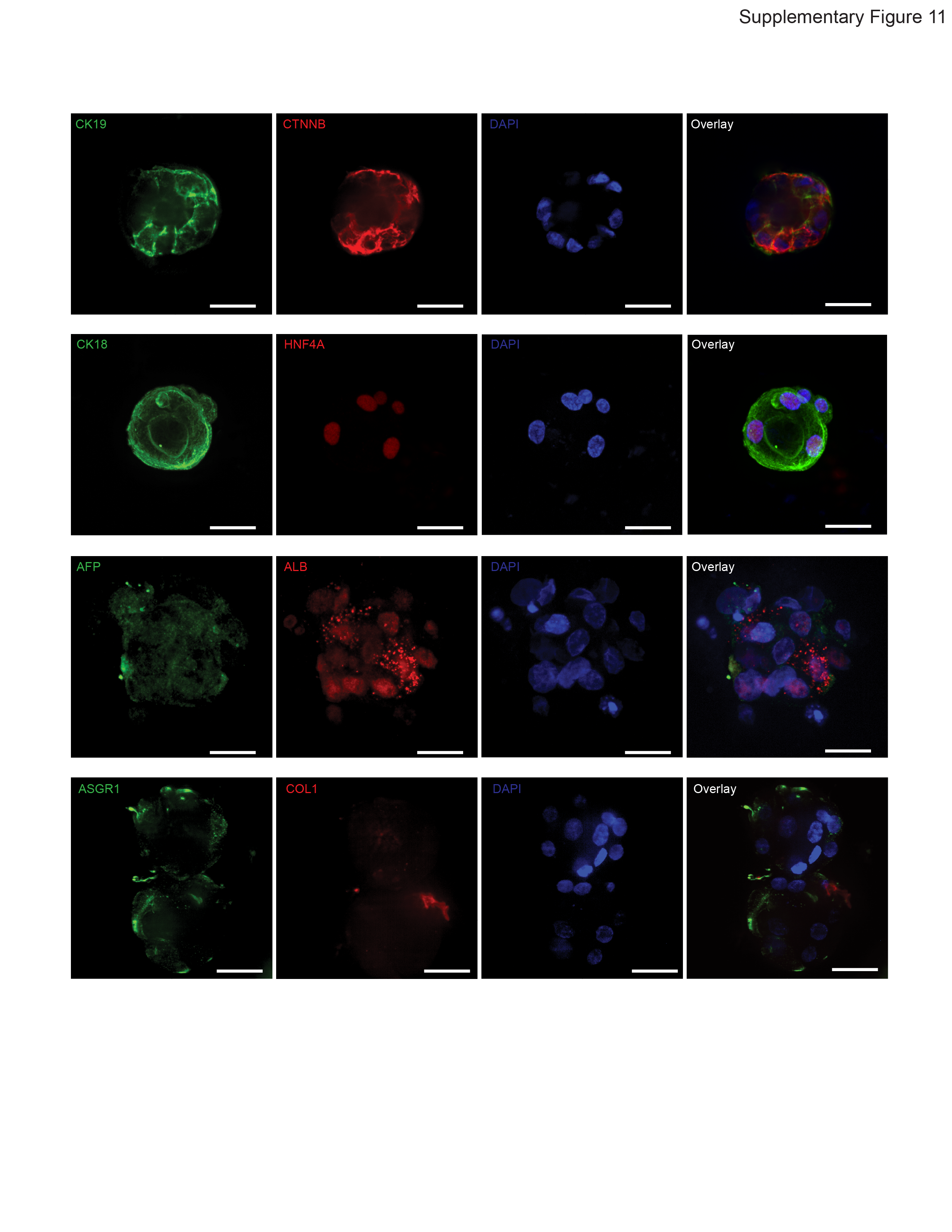
**Supplementary Fig. 11.** Confocal micrographs of IH in Matrigel showing characteristics of organoid-like luminal structure and hepatic markers. Scale bar, 50µm.


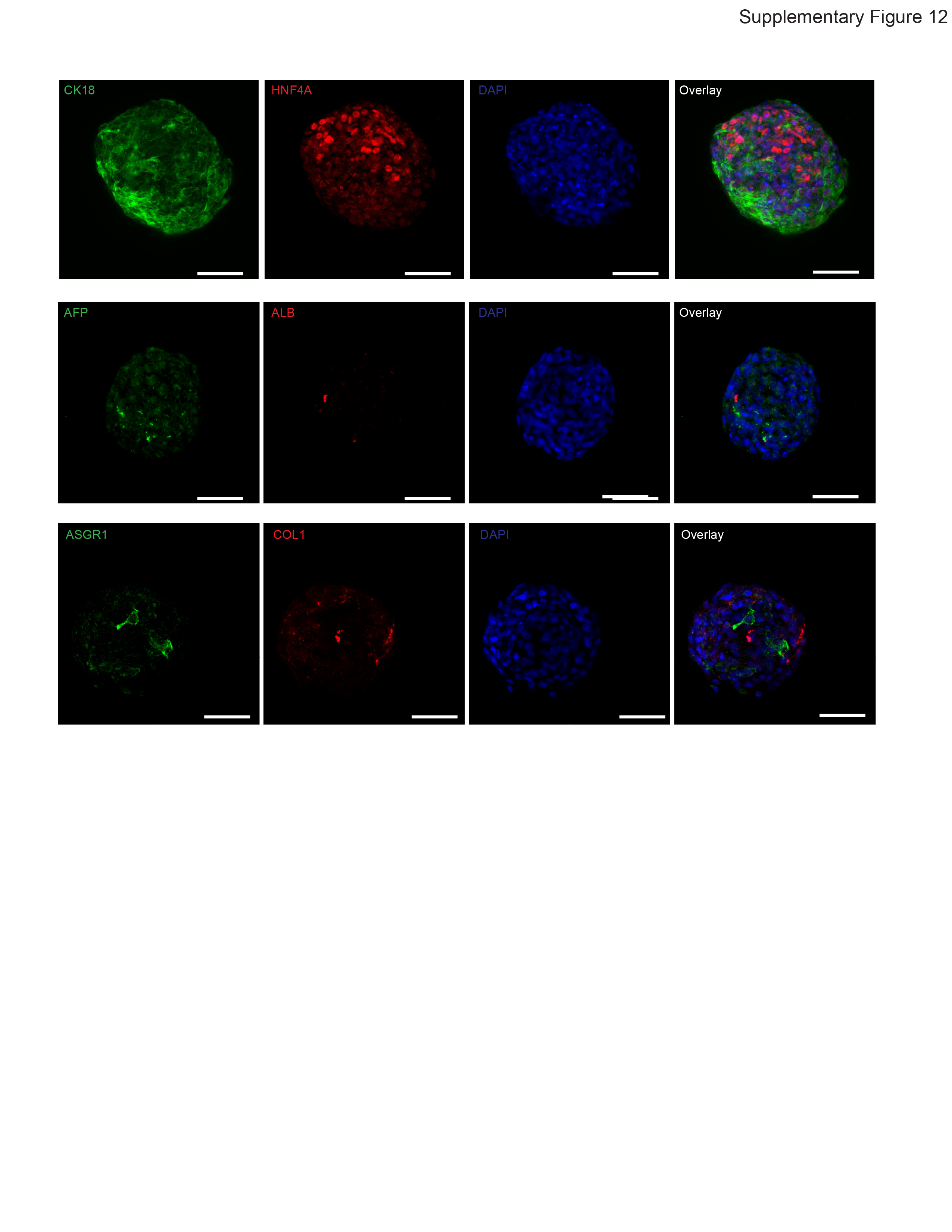
**Supplementary Fig. 12.** Confocal micrographs of IH in spheroids showing characteristics of immature hepatic markers. Scale bar, 100µm.


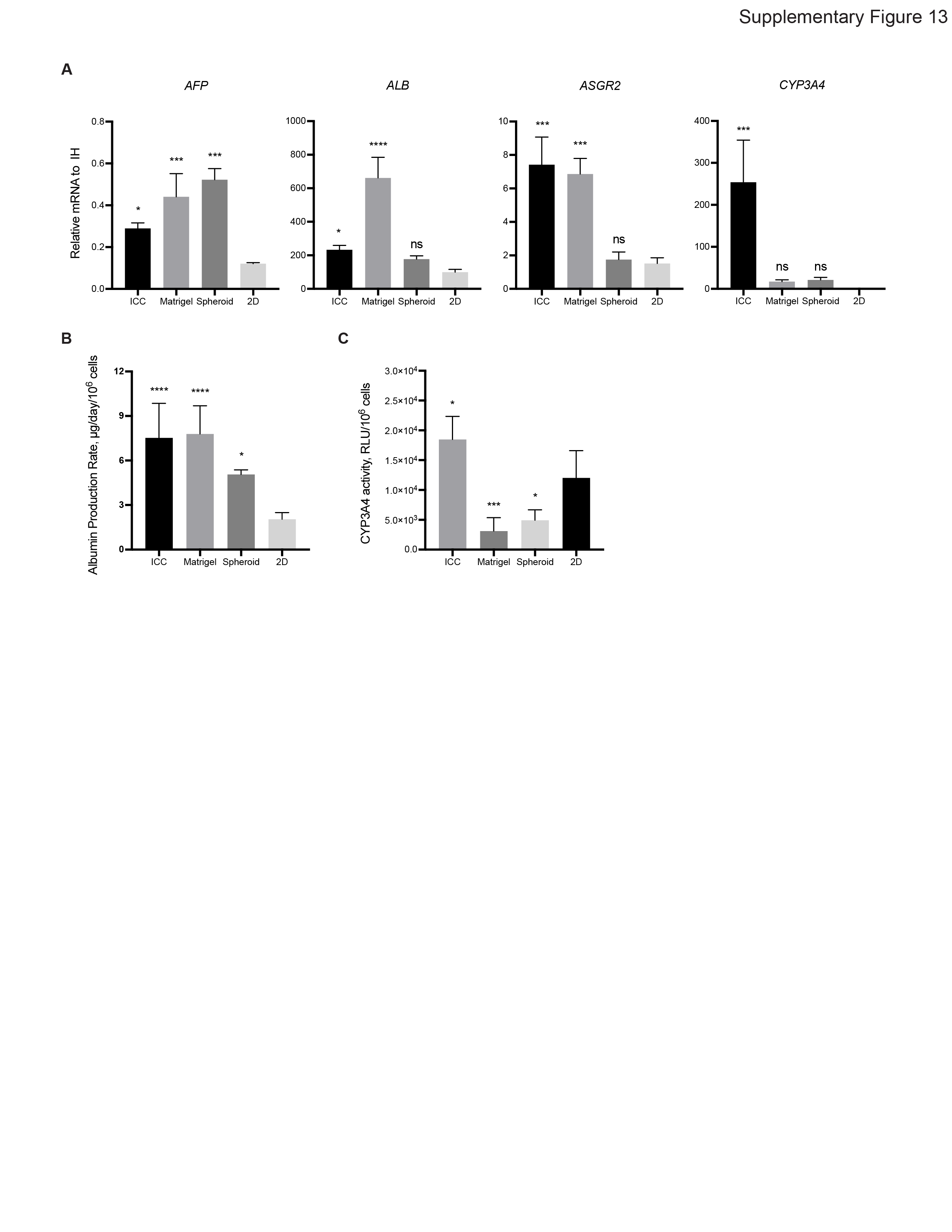


**Supplementary Fig. 13.** IH established organoid in Matrigel and solid spheroids in AggreWell^TM^ exhibit with hepatic phenotypes and functions. (**A**) Differential gene expression (by RT-qPCR) of selected genes reveals a more liver specification of IH in ICC over Matrigel, spheroid and 2D models (N=4). (**B**) Albumin production rate of IH in 2D, ICC, Matrigel and spheroid (N=6). (**C**) Basal metabolic activity of Cytochrome P450 isoform CYP3A4 in IH-ICC, Matrigel, spheroids and 2D. Mean±sd, *p<0.05; **p<0.005; ***p<0.0005; ns nonsignificant.

**
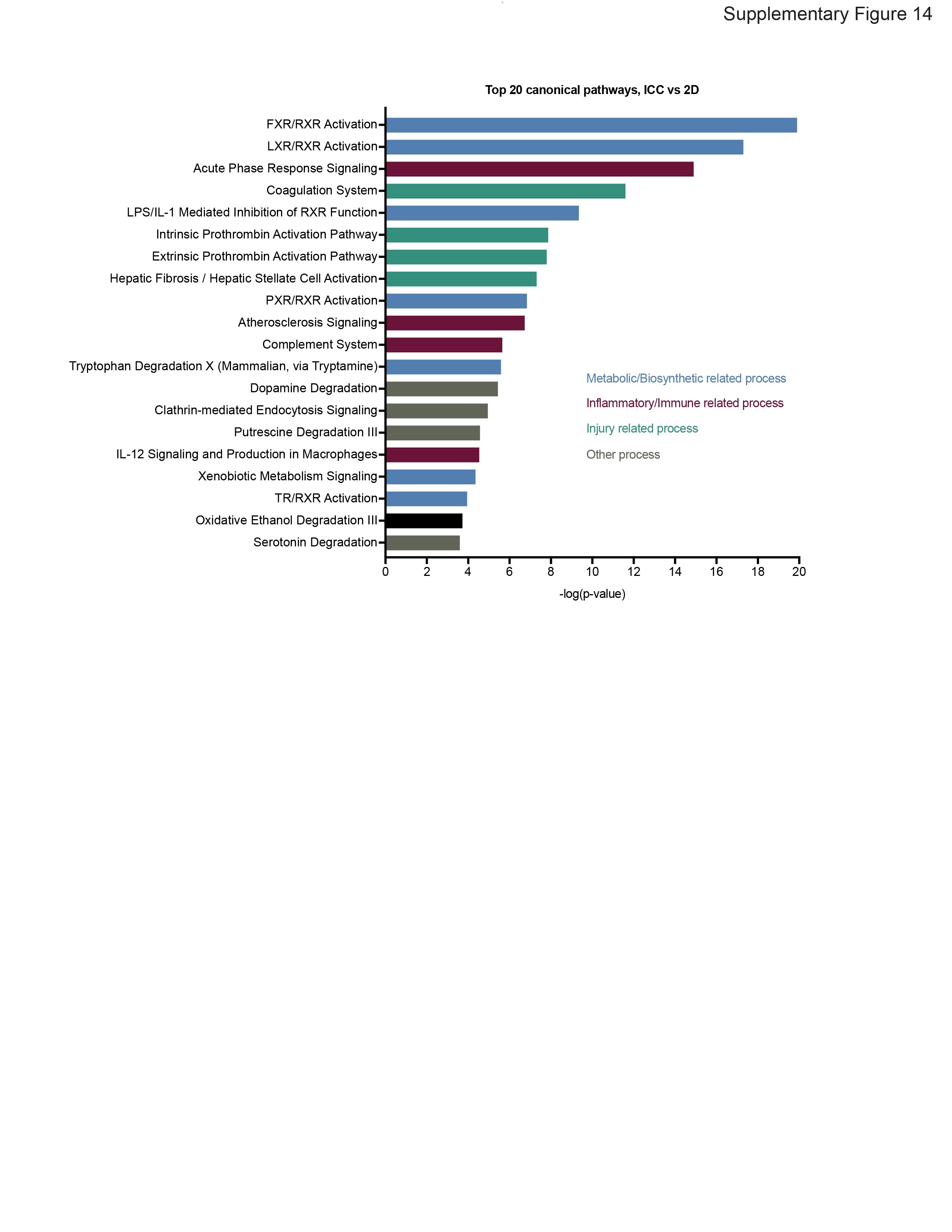
Supplementary Fig. 14.** Ingenuity Pathway Analysis (IPA) revealed the top 20 canonical pathways upregulated in IH-ICC organoids over 2D models.

**
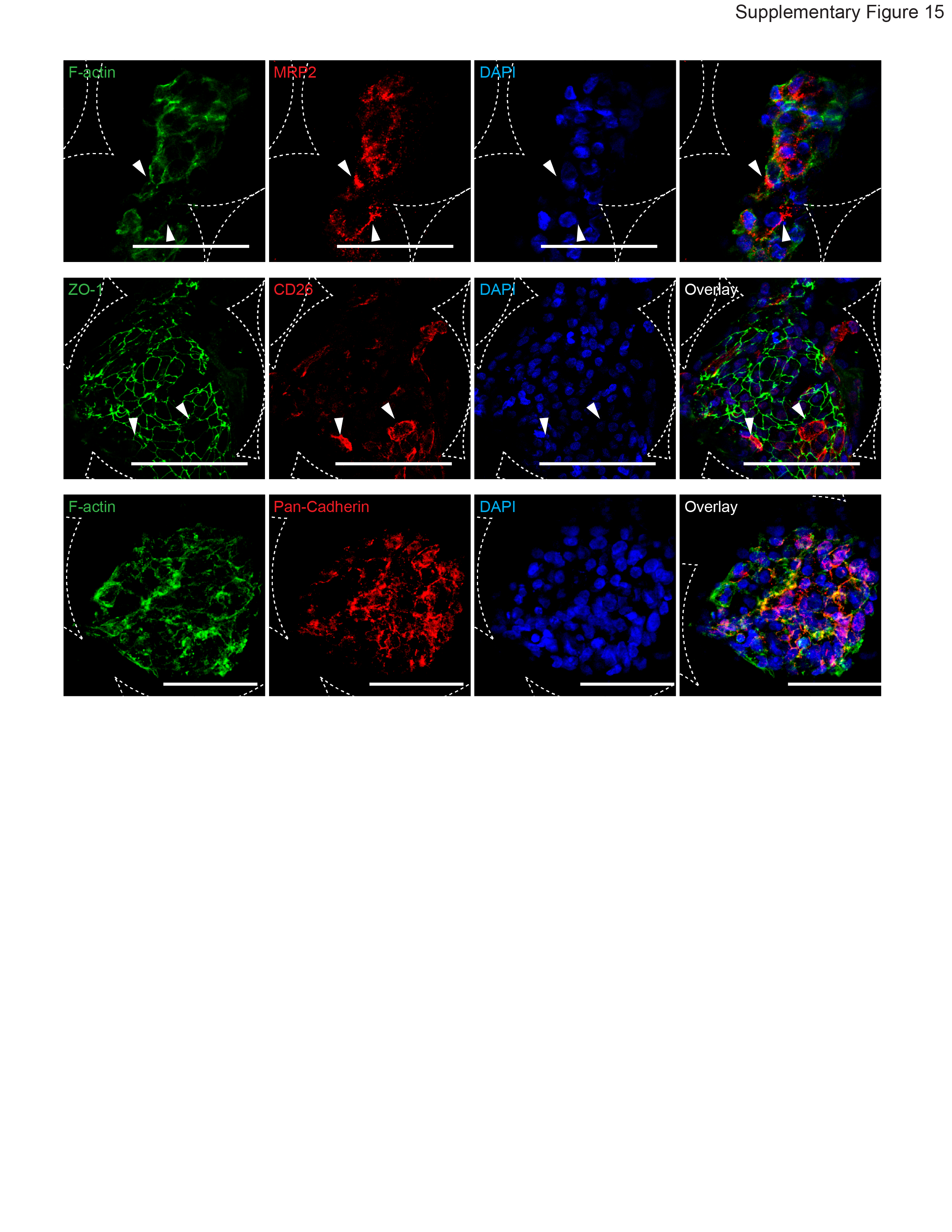
Supplementary Fig. 15.** Single channel and overlay confocal micrographs of images shown in **Fig. 5C**. Scale bar, 100µm. White arrowhead points to apical region.

**
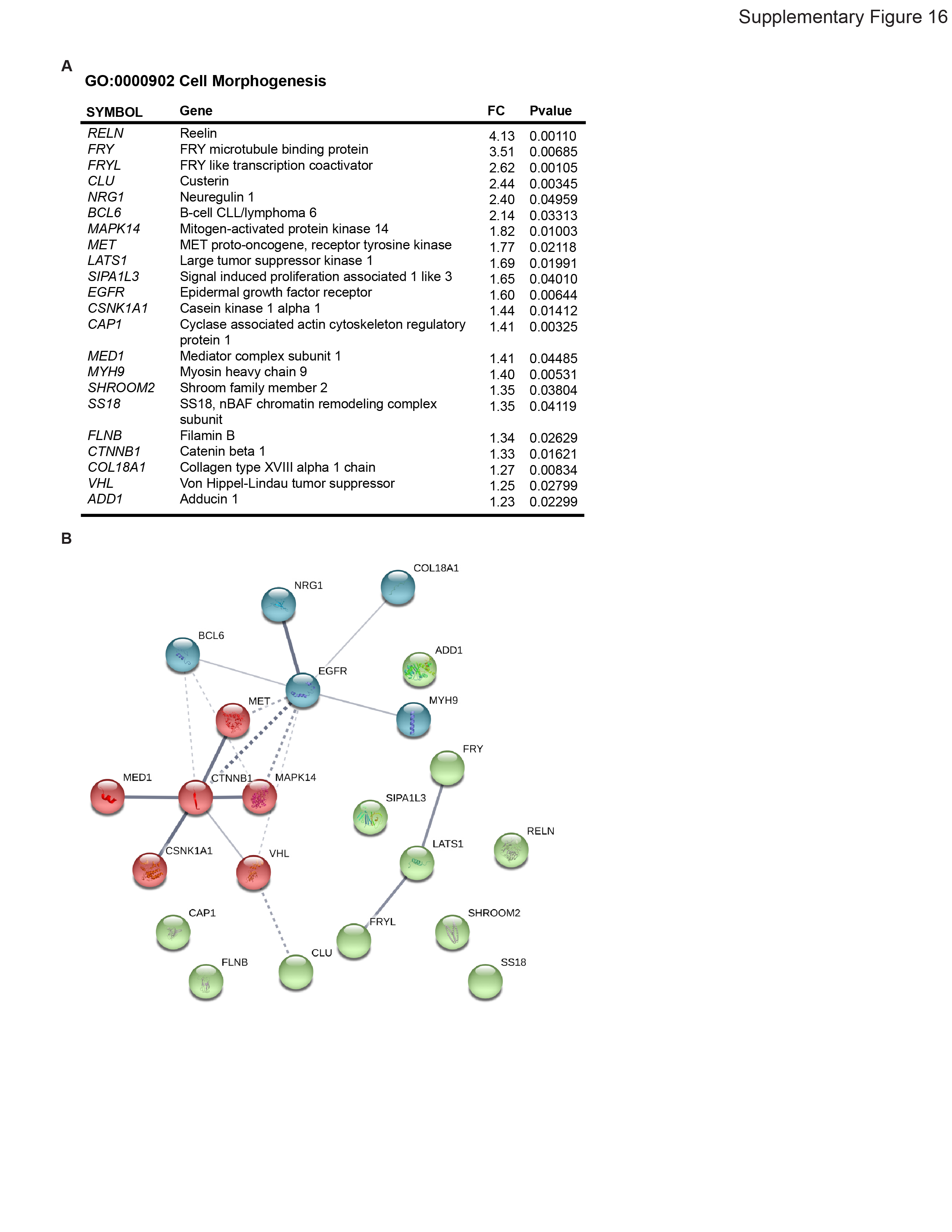
Supplementary Fig. 16.** (**A**) A list of uniquely upregulated genes in IH-ICC vs 2D that involved in cell morphogenesis. FC, fold change. (**B**) The STRING functional network predicted the associations between proteins (nodes) from regulated genes involved in cell polarity in IH-ICC. The cluster analysis was performed using KMEANS clustering algorithms.

**
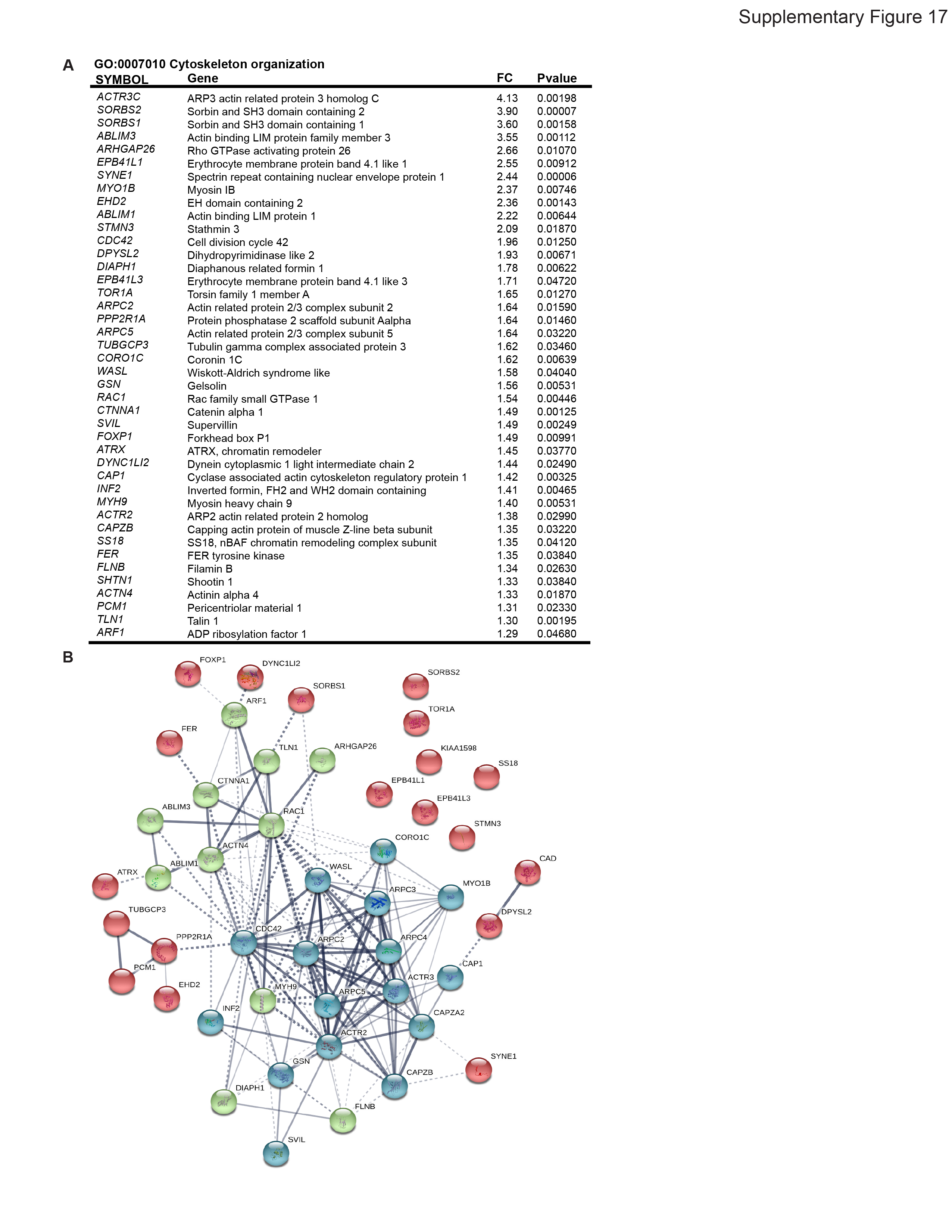
Supplementary Fig. 17.** (**A)** A list of uniquely upregulated genes in IH-ICC vs 2D that involved in cytoskeleton organization. FC, fold change. (**B**) The STRING functional network predicted the associations between proteins (nodes) from regulated genes involved in cell polarity in IH-ICC. The cluster analysis was performed using KMEANS clustering algorithms.

**
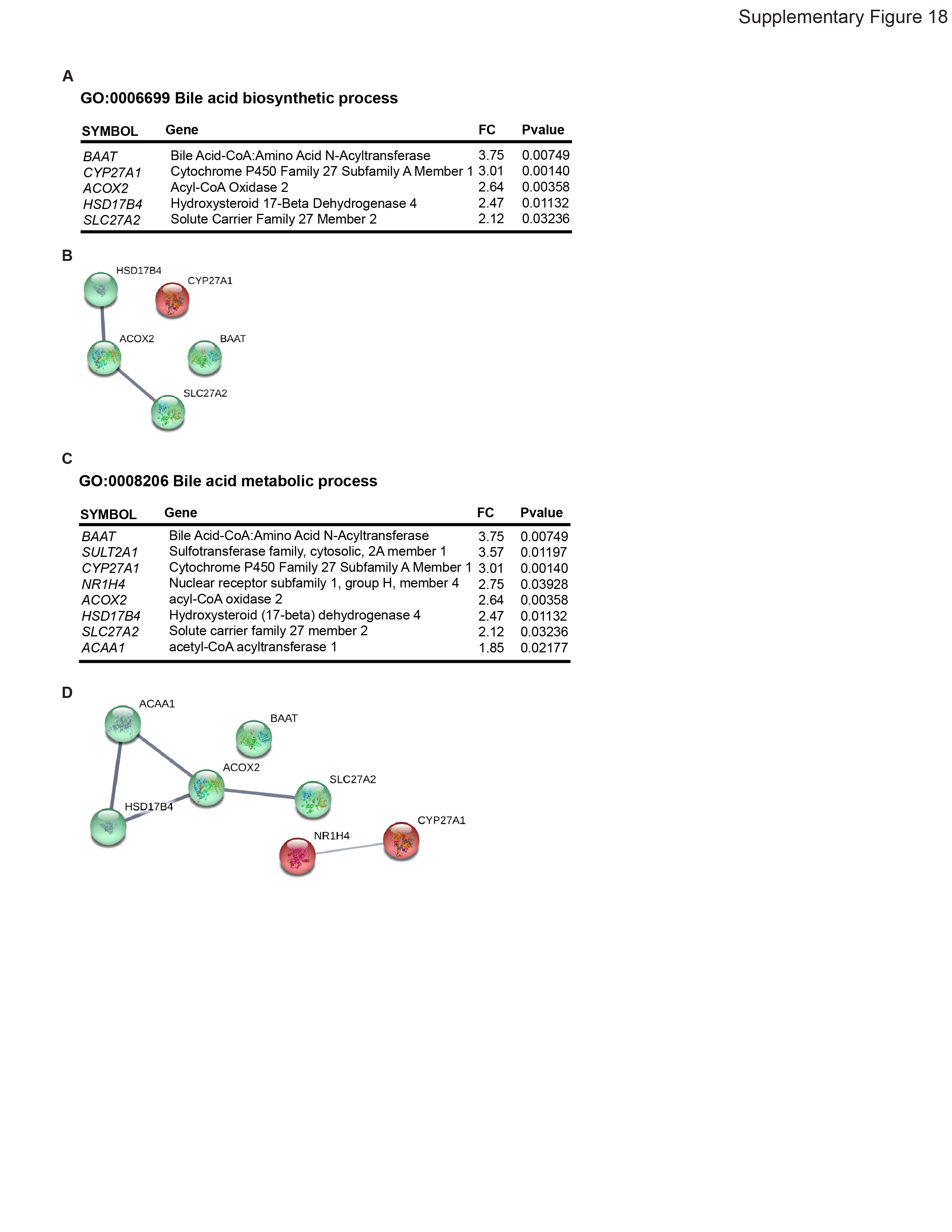
**

**Supplementary Fig. 18.** (**A**) A list of uniquely upregulated genes in IH-ICC vs 2D that involved in bile acid biosynthesis process and (**C**) metabolic process. FC, fold change. (**B** and **D**) The STRING functional network predicted the associations between proteins (nodes) from regulated genes involved in cell polarity in IH-ICC. The cluster analysis was performed using KMEANS clustering algorithms.

**
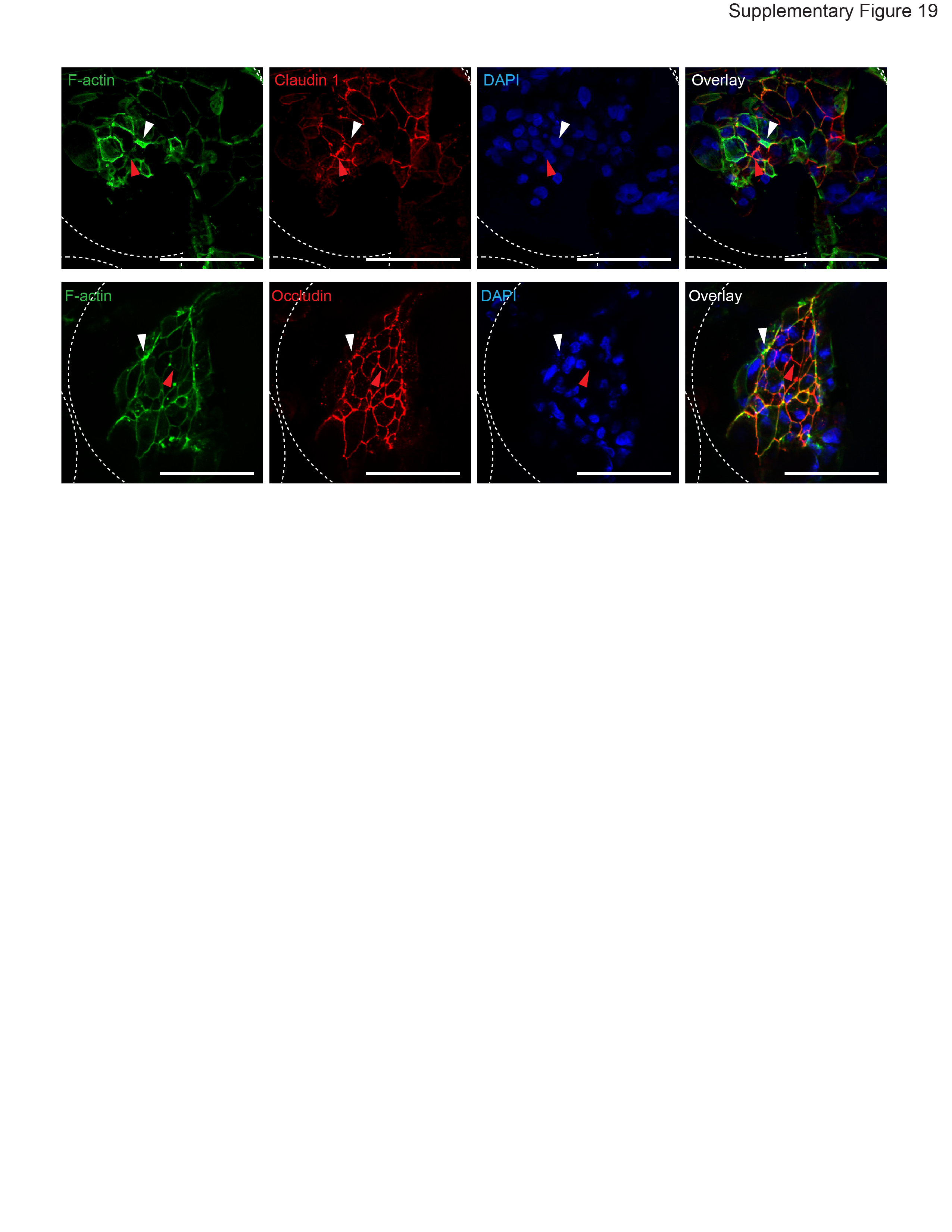
Supplementary Fig. 19.** Single channel and merged confocal micrographs of images shown in **Fig. 6B**. Scale bar, 50µm. White and red arrowheads point to apical and lateral regions respectively.

**
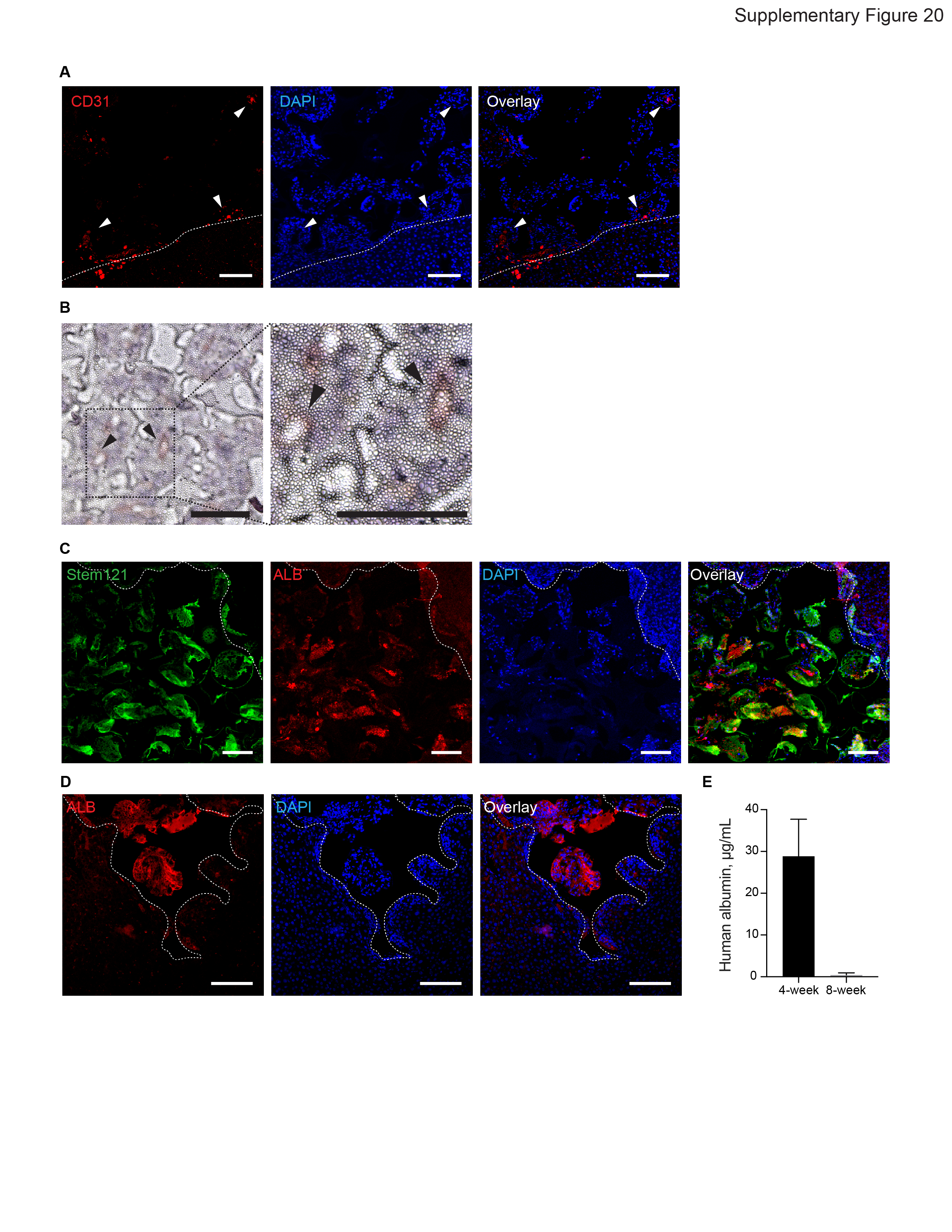
**

**Supplementary Fig. 20.** In vivo transplantation of IH-ICC into immune-compromise mouse. Host endothelial cells lining (CD31 positive cells, indicated by arrowheads) in the explant as shown in immunofluorescence (**A**) and histochemical staining (**B**). IH-ICC expressed mature liver markers in the explant (**C and D**) and produced human albumin (**E**) at 4-week post transplantation. Dashed white line indicates the boundary between implant and host liver. Scale bar, 100µm.

**
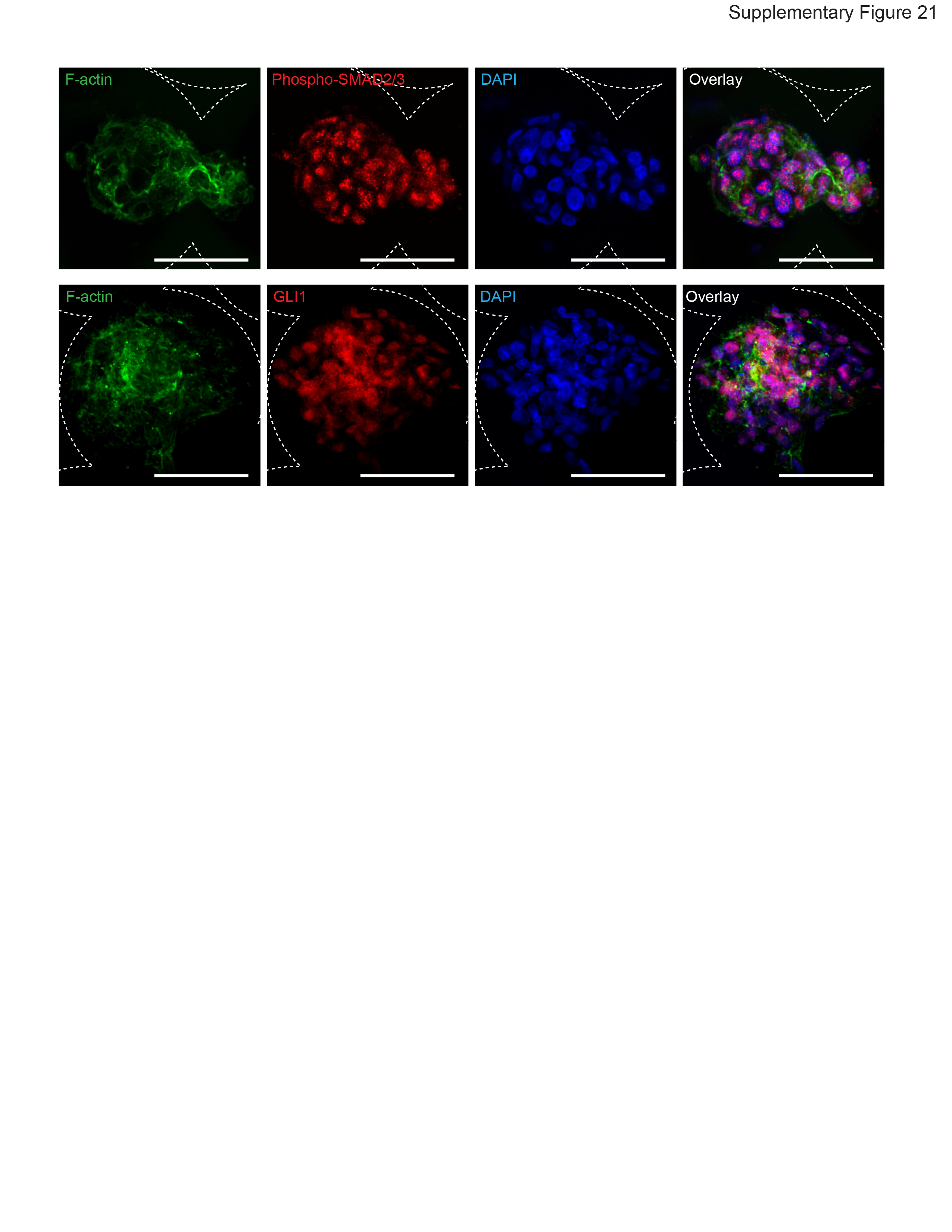
Supplementary Fig. 21.** Single channel and merged confocal micrographs of images shown in Fig. 7E. Scale bar, 50µm.


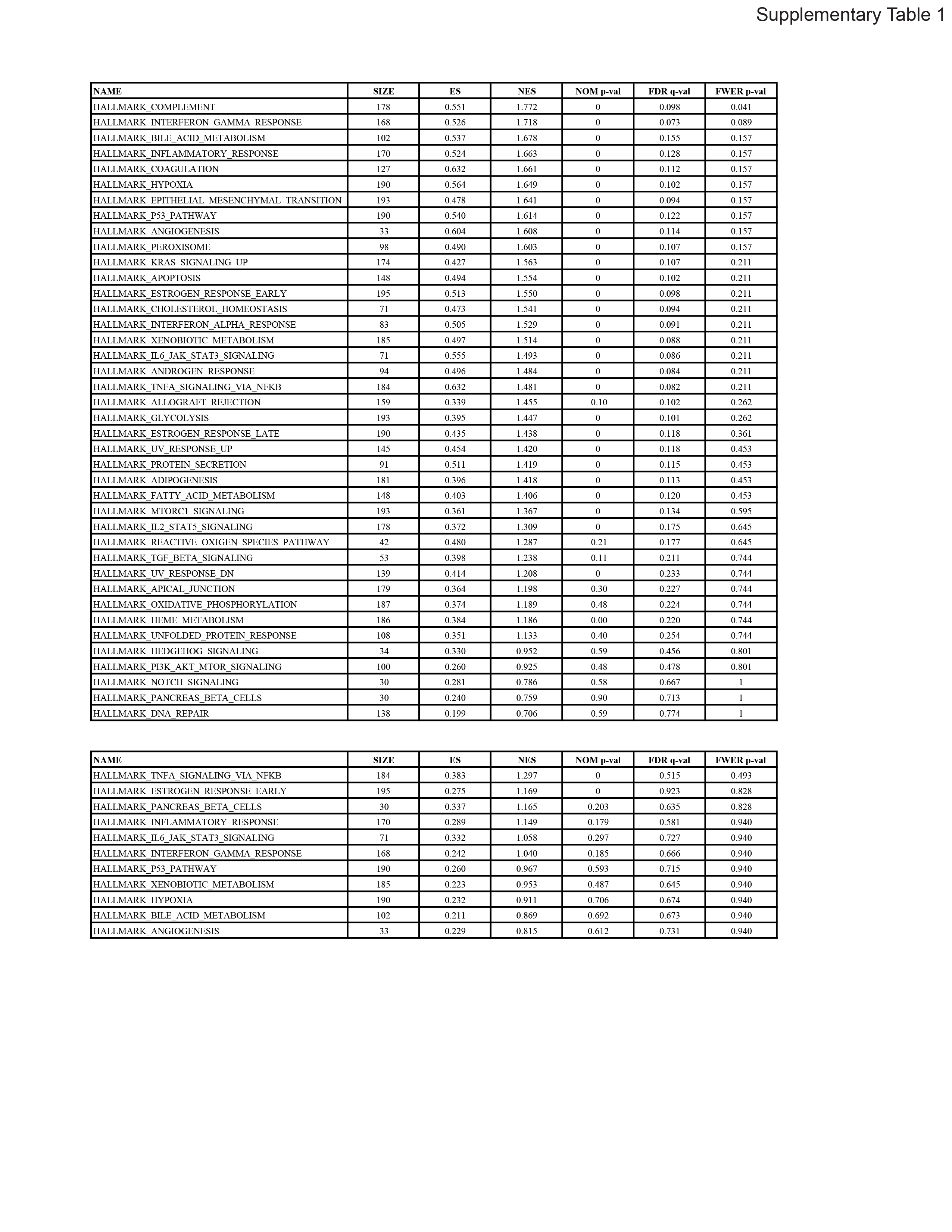


**Supplementary Table 1.** List of gene sets enriched in ICC and 2D over DE.

**
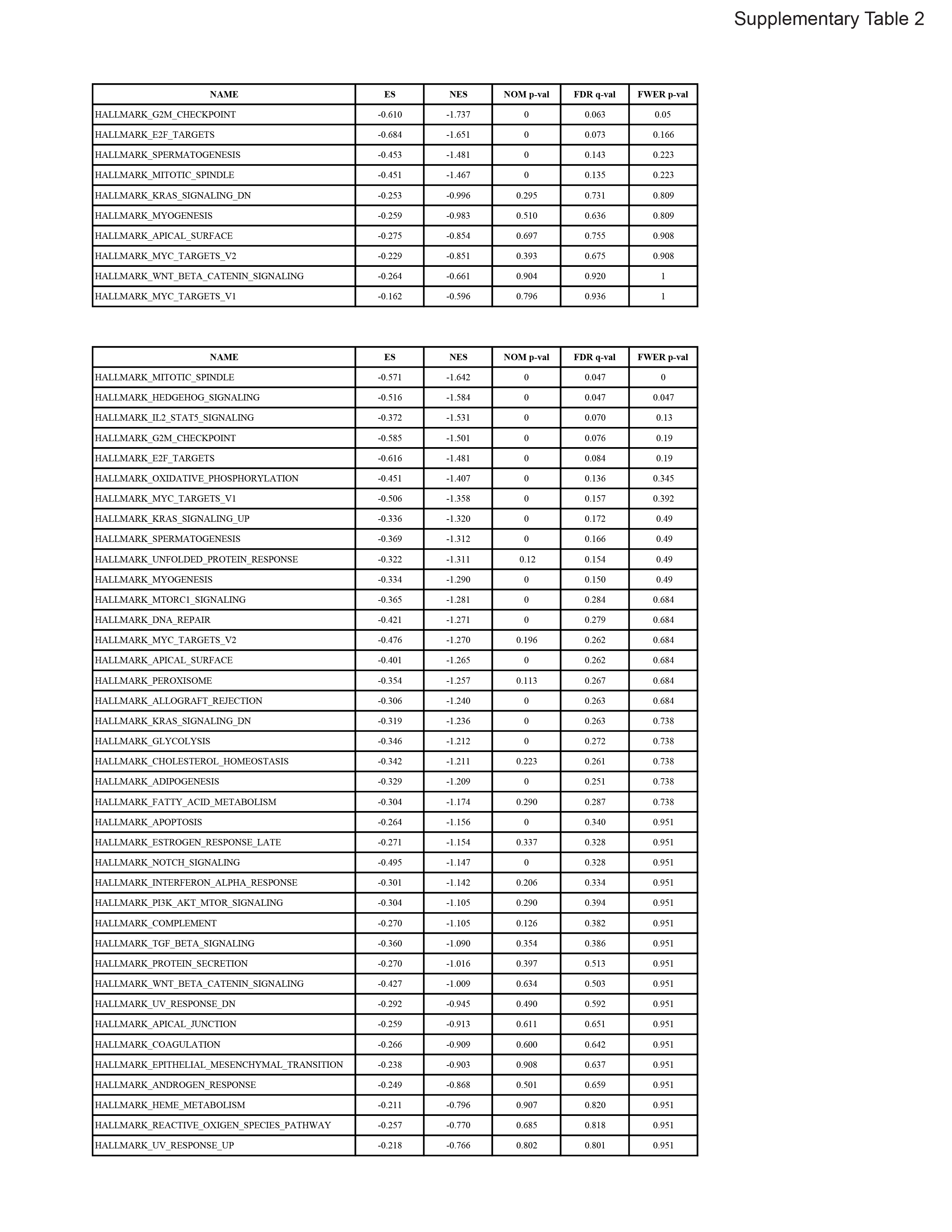
**

**Supplementary Table 2.** List of gene sets dissipated in ICC and 2D over DE.


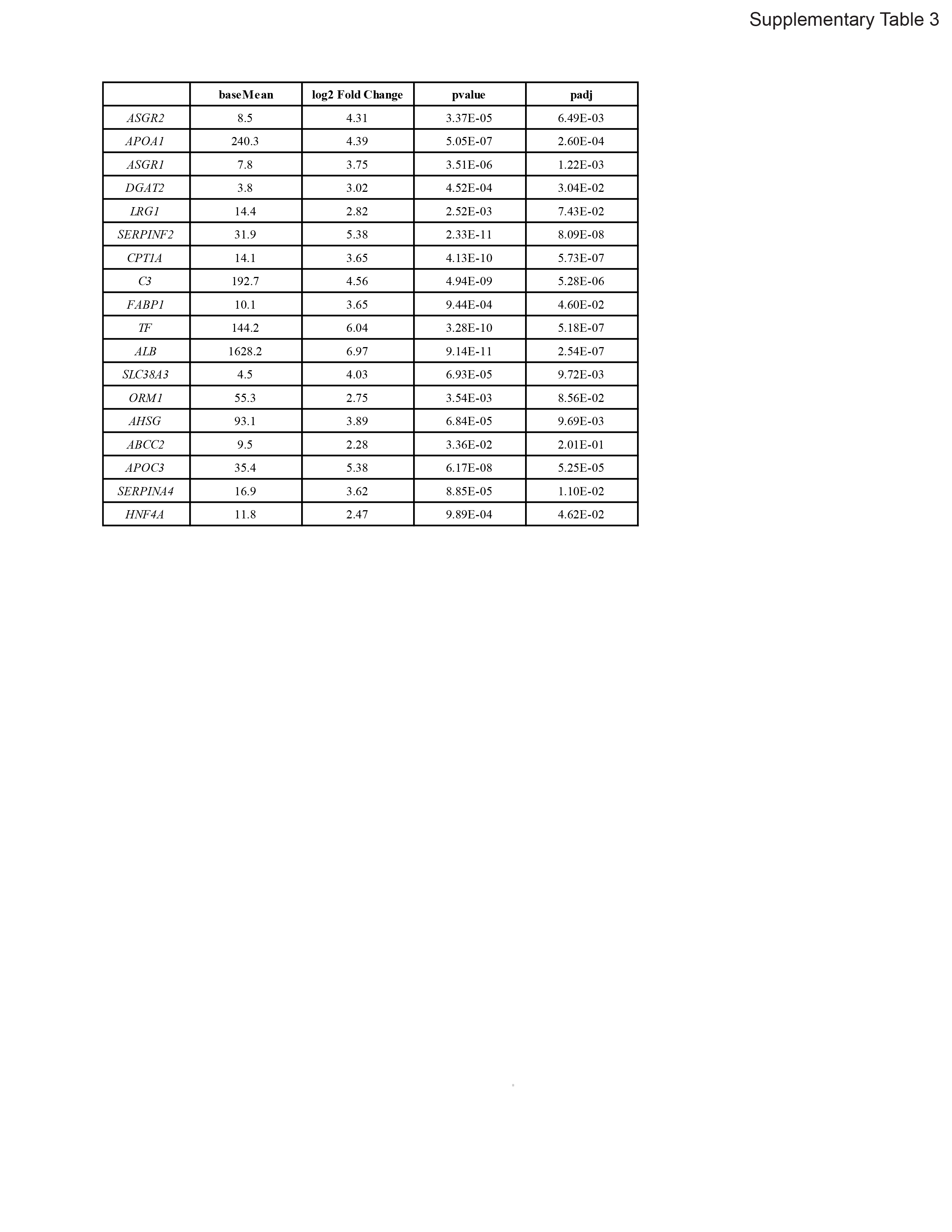


**Supplementary Table 3.** List of top 18 genes unregulated uniquely in ICC over 2D.
